# From Snow Mold Severity to Yield Performance: Identifying Promising Winter Rye Accessions Through Multi-Pathogen and Multi-Trait Assessment

**DOI:** 10.3390/plants15213375

**Published:** 2026-11-01

**Authors:** Mira L. Ponomareva, Sergey N. Ponomarev, Gulnaz S. Mannapova, Irina O. Ivanova, Svetlana Y. Pavlova, Olga A. Gogoleva, Guzalia Sh. Murzagulova, Vladimir Y. Gorshkov

**Affiliations:** 1 Tatar Scientific Research Institute of Agriculture, Federal Research Center “Kazan Scientific Center of the Russian Academy of Sciences”, 420111 Kazan, Russia; 2 Kazan Institute of Biochemistry and Biophysics, Federal Research Center “Kazan Scientific Center of the Russian Academy of Sciences”, 420111 Kazan, Russia

**Keywords:** *Secale cereale*, *Microdochium nivale*, *Typhula*, multi-trait selection, multi-trait genotype–ideotype distance index (MGIDI), winter hardiness

## Abstract

Snow mold is a major constraint to winter cereal production in regions with prolonged snow cover. Because the disease is caused by phylogenetically distinct pathogens with different infection strategies, resistance is commonly treated as a single trait despite limited evidence that host responses are consistent across causal agents. Sixty winter rye accessions were evaluated over two seasons under one natural infectious background and three backgrounds experimentally enriched with *Microdochium nivale*, *Typhula incarnata*, and *T. ishikariensis*. Mean snow mold damage (SMD) increased from 3.69 points under the natural background to 6.54–7.41 points under the three experimentally enriched backgrounds. Accession rankings for SMD differed among backgrounds: rank correlations were weak and mostly non-significant, and the accession × background interaction was significant, indicating background-dependent, possibly partly pathogen-specific responses. Post-winter regenerative capacity was more strongly associated with grain yield than disease severity, highlighting the importance of recovery after snowmelt. The Multi-Trait Genotype–Ideotype Distance Index identified accessions combining favorable disease resistance, winter hardiness, regenerative capacity, and productivity across all backgrounds, while the best-performing accessions under individual backgrounds were identified separately from SMD rankings. These findings support multi-pathogen, multi-trait evaluation for breeding winter cereals with improved adaptation to changing winter environments.

## 1. Introduction

Winter rye (*Secale cereale* L.) is one of the most important cereal crops, with approximately 85% of its global cultivation area concentrated in the agricultural regions of Northern and Central Europe [1]. In 2024, worldwide production of rye reached about 9.25 million tons from roughly 2.76 million hectares across some 60 countries [2]. Rye plays a significant role in food systems, particularly as a staple for bread production, and is also valued for its distinctive flavor in baking and distilling [3,4,5,6]. In addition, its resilience on marginal soils and under low-input conditions makes it a climate-resilient crop of increasing relevance [7,8].

Although rye is generally more stress-tolerant than wheat and barley, stable rye production remains constrained by insufficient winter hardiness. A major cause of winter damage is the development of a disease complex caused by low-temperature fungi that parasitize wintering plants under prolonged snow cover [9]. This complex is collectively referred to as snow mold. It is caused by phylogenetically distant pathogens with distinct modes of interaction with the host, the most important of which are the ascomycete *Microdochium nivale* (pink snow mold) and the basidiomycetes *Typhula ishikariensis* and *T. incarnata* (speckled and gray snow mold, respectively) [10,11]. As these pathogens vary significantly in their biology and infection strategies, resistance identified against one snow mold species may not necessarily provide protection against others. However, resistance to snow mold is still commonly evaluated as a single trait in breeding programs [12,13], and the extent of pathogen specificity remains poorly understood, particularly in winter rye [14].

Snow mold is widespread and economically damaging in eastern Japan, the western United States and Canada, parts of Russia, and Central and Eastern Europe [15,16,17]. In the Pacific Northwest, extended snow cover during the 2016-2017 winter caused substantial crop losses [12]. In Central Russia, snow mold pressure regularly reaches or exceeds the epiphytotic level, and in 2019 the disease led to emergency situations and severe destruction of winter cereal stands in several regions [18]. In this area, *M. nivale* is the dominant snow mold pathogen, although *Typhula*-related snow mold also occurs [19,20,21]. Projected changes in winter climate, including greater variability in snow cover duration and more frequent freeze–thaw events, may alter the distribution and relative importance of snow mold pathogens across winter cereal-growing regions, increasing the need for breeding cultivars with durable resistance to diverse pathogen complexes [22].

Biological control of snow mold remains underdeveloped, despite recent reports of promising antagonistic microorganisms [19,20]. Chemical protection is complicated by the fact that disease development occurs under snow, when fungicide application is impossible, and by the widespread occurrence of fungicide-resistant or less-sensitive strains [23,24,25,26,27]. Consequently, breeding for improved winter hardiness, including resistance to the snow mold complex, represents the most sustainable long-term strategy for disease management [28].

Resistance to snow mold is a polygenic and quantitative trait [29,30]: resistant cultivars show higher survival and yield than susceptible ones, though even the most resistant of them can succumb under sufficiently intense pathogen pressure [18,31]. This quantitative nature of resistance is further complicated by the diversity of snow mold fungi, which differ not only in phylogenetic position but also in survival strategies and host–microbe interactions [11,15,17,32].

Winter crop germplasm remains poorly evaluated under multiple infectious backgrounds enriched with different snow mold pathogens [14,33]. This task is complicated by the fact that the disease is caused by pathogens with different virulence mechanisms and that its severity is strongly influenced by overwintering conditions, making objective phenotyping difficult [34]. In a recent study [35], this problem was partially addressed by evaluating winter rye accessions under natural snow mold pressure and under an experimental background enriched by highly virulent *M. nivale* strains. That work identified promising accessions that are now used both in commercial production and as parental material in breeding programs [35,36]. These findings provided a foundation for investigating the broader biological basis and transferability of snow mold resistance across pathogen complexes. The present study addresses this gap directly by evaluating a largely different germplasm set under a natural infectious background and three separate experimental infectious backgrounds, each enriched with a different major snow mold pathogen.

The objectives of this study were twofold: first, to determine whether resistance of winter rye to snow mold is consistent across pathogen-enriched infectious backgrounds or largely pathogen-dependent, and second, to identify accessions combining favorable levels of disease resistance, winter hardiness, regenerative capacity, and grain yield through an integrated multi-trait evaluation. The evaluation protocol was extended to enable the identification of accessions that maintain high yield after overwintering through different mechanisms and to identify both multi-trait superior accessions and those outstanding for individual economically important traits. Identifying accessions with favorable mean performance across diverse pathogen-enriched backgrounds is particularly important under changing winter climates and provides breeders with a rational basis for developing cultivars with more durable resistance.

## 2. Results

### 2.1. Phenotypic Variability of 60 Winter Rye Accessions Across Infectious Backgrounds

**Figure 1:**
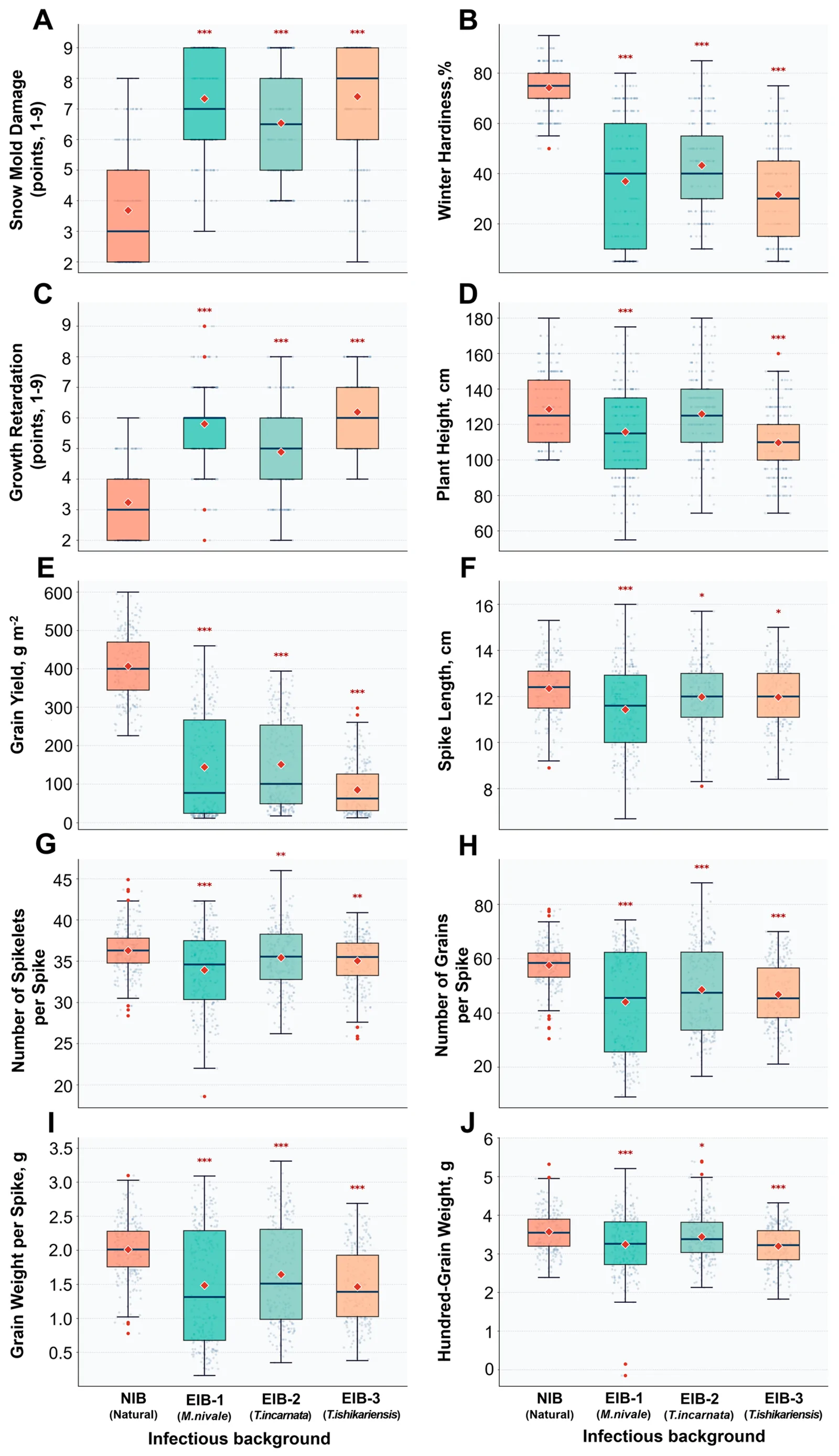
Phenotypic distribution of 10 traits for 60 winter rye (*S. cereale*) accessions grown during the 2023–2024 and 2024–2025 growing seasons across four infectious backgrounds: natural infectious background (NIB), or experimentally enriched infectious backgrounds (EIBs) with *Microdochium nivale* (EIB-1), *Typhula incarnata* (EIB-2), or *Typhula ishikariensis* (EIB-3). The following 10 traits are presented: snow mold damage (**A**), winter hardiness (**B**), growth retardation (**C**), plant height (**D**), grain yield (**E**), spike length (**F**), number of spikelets per spike (**G**), number of grains per spike (**H**), grain weight per spike (**I**), and hundred-grain weight (**J**). Box-and-whisker plots display the median (horizontal line inside the box), the arithmetic mean (red diamond inside the box), quartiles (box height) and the whiskers extending to the most extreme non-outlying values. Asterisks placed above the EIB boxplots indicate that the trait value under the respective EIB differs significantly from that on NIB (Wilcoxon signed-rank test on accession means following a significant Friedman test, Bonferroni-corrected: * *p* < 0.05, ** *p* < 0.01, *** *p* < 0.001).

Figure 1 shows the distribution of ten agronomic and disease-related traits for the 60 winter rye accessions across the four infectious backgrounds: (1) natural infectious background (NIB); (2) experimentally enriched infectious background (EIB) with *M. nivale* (hereafter EIB-1); (3) EIB with *T. incarnata* (hereafter EIB-2); and (4) EIB with *T. ishikariensis* (hereafter EIB-3). All 10 traits showed pronounced differences among infectious backgrounds (Friedman test, χ^2^ = 32.3–153.0, df = 3, *p* < 0.001 for all traits), indicating that the phenotypic expression of the traits studied depends on the type of infectious background.

#### 2.1.1. Snow Mold Damage (SMD)

Under NIB, mean SMD was 3.69 ± 0.87 points (CV = 23.5%), ranging from 2.00 to 6.25 points (Figure 1A, Table S1). Under EIBs, SMD increased substantially compared to NIB, while phenotypic variability among the studied accessions decreased. Mean SMD was 7.34 ± 0.60 under EIB-1 (*M. nivale*), 6.54 ± 0.57 under EIB-2 (*T. incarnata*), and 7.41 ± 0.64 under EIB-3 (*T. ishikariensis*). These values correspond to increases of approximately 2.9–3.7 SMD points relative to NIB. Phenotypic variability was lower under the three EIBs relative to NIB, as indicated by lower coefficients of variation (CV = 8.1–8.7% vs. 23.5% under NIB), reflecting reduced phenotypic dispersion at the higher disease levels observed under the EIBs. Because mean SMD under EIB-1 and EIB-3 approached the upper end of the 1–9 scale, the lower variability under these backgrounds may partly reflect a ceiling effect of the scoring scale.

#### 2.1.2. Winter Hardiness

Mean winter hardiness (WH) under NIB was 74.21 ± 5.80% (CV = 7.8%), ranging from 61.25 to 85.00% (Table S1) (Figure 1B). WH declined substantially under EIBs. The strongest reduction was observed under EIB-3 (*T. ishikariensis*), where mean WH was 31.60 ± 8.09% (a 57.4% decrease relative to NIB; CV = 25.6%). Under EIB-1 (*M. nivale*), mean WH was 36.96 ± 8.15% (−50.2%; CV = 22.0%), and under EIB-2 (*T. incarnata*) it was 43.27 ± 8.25% (−41.7%; CV = 19.1%). Compared with NIB, coefficients of variation increased markedly under EIBs (19.1–25.6%), indicating greater phenotypic variation among accessions under the pathogen-enriched backgrounds.

#### 2.1.3. Growth Retardation

Post-winter growth retardation (GR) under NIB was relatively moderate (mean = 3.23 ± 0.65 points) (Figure 1C, Table S1). Under EIBs, GR was substantially impaired. The strongest retardation was observed under EIB-3 (*T. ishikariensis*), where mean GR reached 6.19 ± 0.62 points (+91.5% relative to NIB; CV = 10.0%). The low coefficient of variation under EIB-3 indicated that nearly all accessions recovered poorly and to a fairly uniform degree. Under EIB-1 (*M. nivale*), mean GR was 5.80 ± 0.80 points (+79.5%), and under EIB-2 (*T. incarnata*) it was 4.89 ± 0.94 points (+51.2%) (Figure 1C).

#### 2.1.4. Plant Height

Mean plant height (PH) under NIB was 128.62 ± 17.45 cm (CV = 13.6%), ranging from 102.50 to 167.50 cm (Figure 1D, Table S1). PH declined under all EIBs, though to a variable extent. The strongest reduction was observed under EIB-3 (*T. ishikariensis*), where mean PH was 109.85 ± 15.07 cm (−14.6% relative to NIB; CV = 13.7%). Under EIB-1 (*M. nivale*), mean PH was 115.88 ± 14.12 cm (−9.9%; CV = 12.2%), while under EIB-2 (*T. incarnata*), the reduction was smallest, with mean PH of 125.98 ± 16.49 cm (−2.1%; CV = 13.1%) (Figure 1D).

#### 2.1.5. Grain Yield and Yield Components

Under NIB, grain yield ranged from 298.50 to 539.50 g m^−2^, with a mean of 407.26 ± 67.08 g m^−2^ (Table S1). Under EIBs, substantial yield losses occurred across all treatments: 64.6% under EIB-1 (144.31 ± 42.84 g m^−2^), 62.8% under EIB-2 (151.46 ± 41.27 g m^−2^), and 79.0% under EIB-3 (85.53 ± 27.59 g m^−2^). The coefficient of variation increased from 16.5% under NIB to 27.2–32.3% under EIBs, reflecting greater phenotypic variation among accessions under the pathogen-enriched backgrounds.

Among yield components, spike length (SL) and the number of spikelets per spike (NSS) remained relatively stable across infectious backgrounds. Mean SL was 12.34 ± 1.01 cm under NIB versus 11.44–11.98 cm under EIB-1–EIB-3 (a 2.9–7.3% decrease); the CV for NSS ranged from 5.6% to 6.4% across all infectious backgrounds. In contrast, the number of grains per spike (NGS) showed moderate sensitivity to high infection pressure, decreasing by an average of 15.6–23.5% under EIBs compared with NIB. Grain weight per spike (GWS) declined even more sharply under EIBs (18.2–27.2%). Hundred-grain weight (HGW) showed a smaller decrease under EIBs (3.5–10.4%). The greatest phenotypic variability was observed for GWS under EIB-3 (CV = 22.5%), indicating substantial variation among accessions in the ability to maintain grain productivity (Figure 1E–J).

### 2.2. Snow Mold Resistance Patterns of Rye Accessions Across Infectious Backgrounds and Identification of Background-Specific Resistant Accessions

Within each infectious background, accession had a significant effect on all ten traits (Table S2). A combined ANOVA across all four backgrounds and both years confirmed a highly significant accession main effect for every trait (Table S3). Differences among backgrounds were significant for all traits (Friedman test; Section 2.1, Figure 1). Pairwise Wilcoxon signed-rank tests confirmed that SMD under each of the three EIBs differed significantly from that under NIB (*p* < 0.001). Among EIBs, EIB-2 (*T. incarnata*) induced significantly lower SMD (*p* < 0.001) than either EIB-1 (*M. nivale*) or EIB-3 (*T. ishikariensis*), whereas EIB-1 and EIB-3 did not differ significantly from each other in this respect (Figure 1A). Across backgrounds, accession means for SMD were significantly correlated with WH (ρ = −0.494), GR (ρ = +0.501), and GY (ρ = −0.512) (Table S4), indicating that differences in disease severity were associated with overwintering and productivity; these relationships are examined in detail in Section 2.3.

No accession reached the resistant category (SMD ≤ 3.0) under any of the EIBs, whereas 25.0% did so under NIB, confirming the higher disease pressure of EIBs (Table 1). Under EIB-1 (*M. nivale*), 66.7% of accessions were classified as susceptible, followed by EIB-3 (*T. ishikariensis*) with 63.3% and EIB-2 (*T. incarnata*) with 11.7% (Table 1). Under EIB-2, the majority of accessions (86.7%) fell into the moderately susceptible class, indicating a narrower range of responses under this background.

Under NIB, Grafinya showed the lowest snow mold damage (SMD = 2.0). Four accessions (Chulpan 9, D-3918, Kiprez, and I-30/88) scored 2.5, and another group (Bezenchukskaya 110, Getera 2, Volkhova 2, Evrika, Ritm) scored 2.8 (Table 2). Under EIB-1 (*M. nivale*), Estafeta Tatarstana and Ritm were the top performers (SMD = 6.0), followed by Nemchinovskaya 1 and Kiprez (SMD = 6.3). Under EIB-2 (*T. incarnata*), D-3918 had the best score (SMD = 5.0), followed by Tatarskaya 1 (SMD = 5.5) and a group of four accessions (Getera 2, Arga, Irina, and Ophelia) with SMD = 5.8. Under EIB-3 (*T. ishikariensis*), the leading accessions were Vostok and D-10392 (SMD = 6.0), followed by Bezenchukskaya 110, Irina, and Lota (SMD = 6.3). Notably, the accessions with the lowest SMD differed substantially across backgrounds. This was statistically supported by weak-to-moderate, often non-significant, Spearman rank correlations (ρ) of SMD between pairs of backgrounds (ρ = −0.05 to 0.43; only one of the six pairs remained significant after Holm correction for multiple testing, Table S5). Together with the significant accession × background interaction for SMD in the combined ANOVA (F = 1.63, *p* < 0.001; Table S3), this indicates that accession responses depended on the infectious background, consistent with partly pathogen-specific resistance; however, background effects cannot be fully separated from nursery effects.

No accession ranked among the most resistant under both NIB and all three EIBs. Several accessions were among the top performers under two backgrounds: Kiprez and Ritm ranked among the best under both NIB and EIB-1; D-3918 and Getera 2 combined good resistance under NIB and EIB-2; and Bezenchukskaya 110 performed well under NIB and EIB-3 (Table 2).

A combined ANOVA across all four backgrounds and both years (Table S3) confirmed a highly significant accession main effect for every trait (*p* < 0.001), corroborating the within-background results (Table S2). The accession × background interaction was significant for SMD, GR, PH, SL, NSS, and HGW (all *p* < 0.05), but not for WH, GY, NGS, or GWS. This indicates that, while absolute trait levels shifted markedly among backgrounds (Section 2.1), the relative ranking of accessions for disease severity and certain yield-structure traits changed significantly among backgrounds, consistent with the weak inter-background Spearman correlations for SMD (Table S5). Because these tests are conservative (Section 4.6), the absence of a significant interaction for the remaining traits is not interpreted as evidence of stable accession ranking. The accession × year interaction was significant for six of the ten traits (SMD, GR, PH, GY, NSS, HGW; *p* < 0.05), reflecting the contrasting weather conditions of the two growing seasons (Table 3).

### 2.3. Relationships Between Snow Mold Damage, Productivity, and Spike Architecture

**Figure 2:**
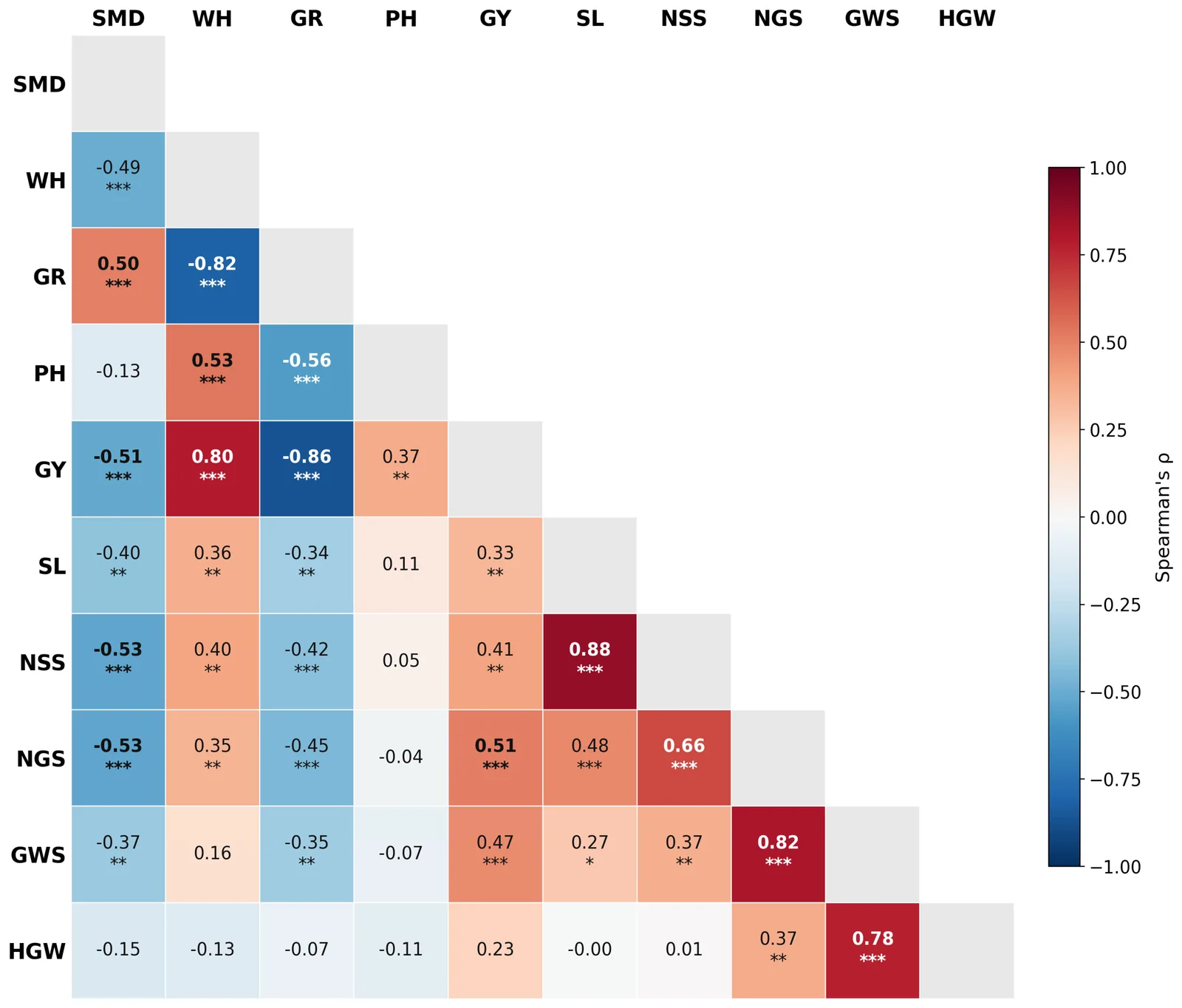
Spearman correlations between traits for 60 winter rye accessions grown during the 2023–2024 and 2024–2025 growing seasons across four infectious backgrounds: natural infectious background (NIB), or experimentally enriched infectious backgrounds (EIBs) with *Microdochium nivale* (EIB-1), *Typhula incarnata* (EIB-2), or *Typhula ishikariensis* (EIB-3). The following 10 traits were assessed: snow mold damage (SMD), winter hardiness (WH), growth retardation (GR), plant height (PH), grain yield (GY), spike length (SL), number of spikelets per spike (NSS), number of grains per spike (NGS), grain weight per spike (GWS), and hundred-grain weight (HGW). Asterisks indicate the significance of the correlations: * *p* < 0.05, ** *p* < 0.01, *** *p* < 0.001.

Correlations were calculated from accession means averaged across the four infectious backgrounds; they therefore describe associations among mean performances and should not be assumed to hold within each individual background. SMD showed significant negative correlations with WH (ρ = −0.494), GY (ρ = −0.512), NGS (ρ = −0.527), NSS (ρ = −0.529), and SL (ρ = −0.400), together with a significant positive correlation with GR (ρ = +0.501) (Figure 2, Table S4). These associations indicate that higher SMD was negatively associated with plant survival and yield, as well as with fewer grains and spikelets per spike.

The strongest negative correlation was observed between GR and GY (ρ = −0.862) (Table S4), indicating that accessions with better post-winter regeneration tended to have higher yields. GR remained significantly associated with GY across all backgrounds (β = −0.20 to −0.49, *p* < 0.001), whereas the association of SMD with GY was weak and non-significant in three of the four backgrounds (β = −0.02 to +0.07). WH showed a strong negative correlation with GR (ρ = −0.819) and a strong positive correlation with GY (ρ = +0.796), indicating that higher yields were associated with both better overwintering and more rapid spring regrowth.

PH showed a moderate positive correlation with WH (ρ = +0.534) and a negative correlation with GR (ρ = −0.555), indicating that taller accessions tended to combine better WH with lower GR scores (i.e., more complete post-winter recovery). HGW showed no significant correlations with either SMD (ρ = −0.151) or GR (ρ = −0.066), indicating that grain size was largely independent of disease severity and regeneration rate.

### 2.4. Principal Component Analysis (PCA) of Resistance–Productivity Relationships in the Studied Winter Rye Germplasm

PCA identified eight components with eigenvalues ≥ 1 that jointly explained 81.2% of the total phenotypic variance (Table S6). PC1 represented the primary resistance–productivity axis. The largest positive loadings on PC1 were contributed by GY and WH across EIBs (GY_EIB-1: +0.238; GY_EIB-2: +0.227; WH_EIB-2: +0.204), whereas GR and SMD under EIBs loaded strongly and negatively (GR_EIB-2: −0.230; GR_EIB-1: −0.209; SMD_EIB-1: −0.202; Table S7). This polarity reflects the fundamental antagonism between disease susceptibility and productive capacity: accessions with high PC1 scores combined better WH and GY under stress with lower disease severity and rapid post-winter recovery. The continuous distribution of accession scores along PC1 is consistent with a quantitative distribution of resistance rather than discrete classes; however, it does not by itself exclude the existence of such classes.

PC2 captured a secondary axis of variation linking grain characteristics with plant height. Positive loadings were contributed by GWS (NIB: +0.319; EIB-2: +0.267), NGS (NIB: +0.261; EIB-2: +0.213), and HGW (NIB: +0.254; EIB-2: +0.241), whereas PH had negative loadings (NIB: −0.283; EIB-1: −0.256; EIB-3: −0.216). In other words, beyond the main resistance–productivity gradient, a second dimension separated shorter accessions with higher grain weight from taller accessions with lower grain weight.

**Figure 3:**
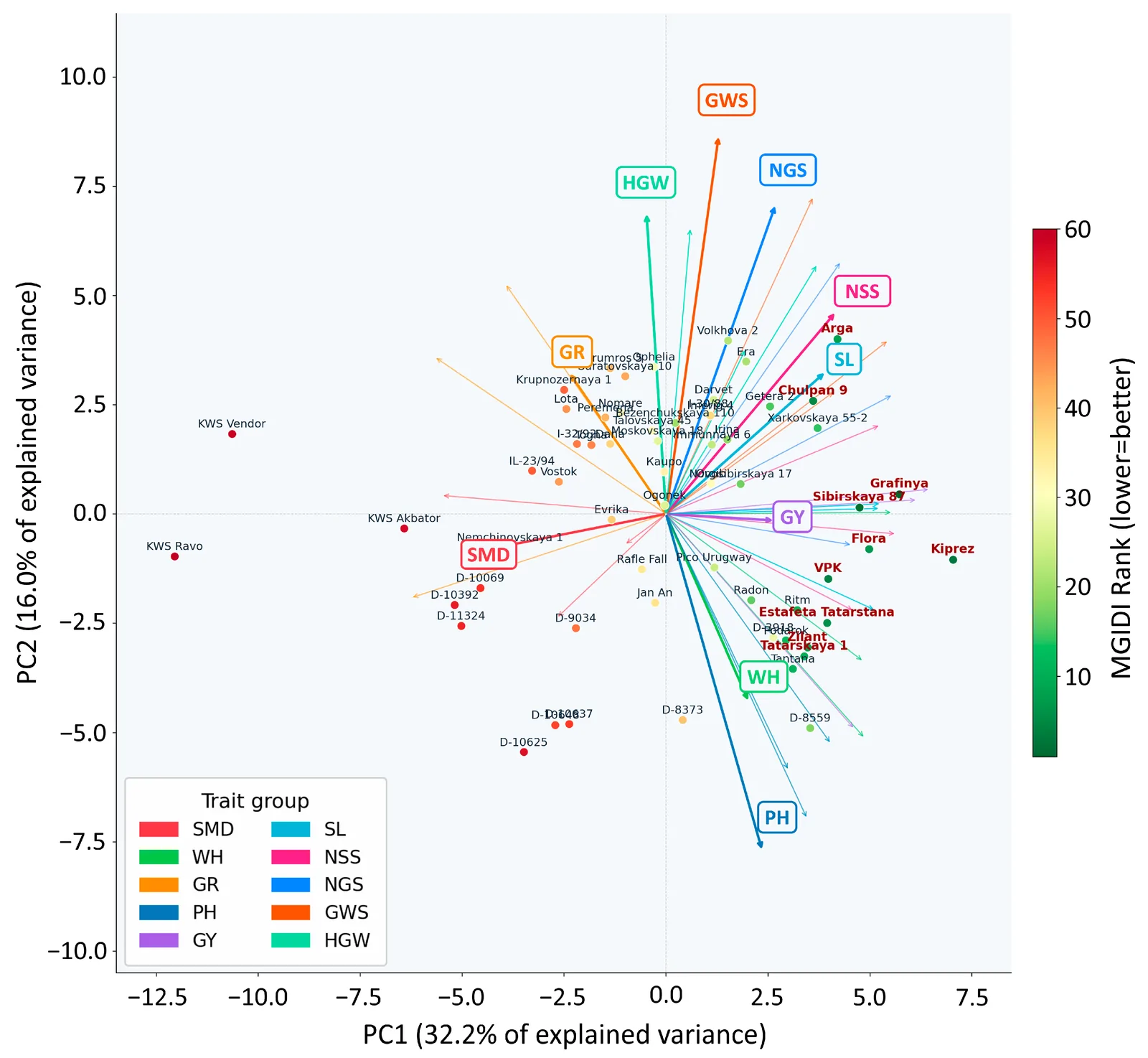
Principal component analysis (PCA) biplot of phenotypic variation among 60 winter rye (*S. cereale*) accessions across four infectious backgrounds: natural infectious background (NIB), or experimentally enriched infectious backgrounds (EIBs) with *Microdochium nivale* (EIB-1), *Typhula incarnata* (EIB-2), or *Typhula ishikariensis* (EIB-3). Circles represent accession scores colored by the multi-trait genotype–ideotype distance index (MGIDI) rank (green: rank 1, best; red: rank 60, worst); bold labels indicate top-10 performers. Arrows indicate trait loadings: thick lines—NIB, thin semi-transparent lines—EIBs 1–3. Trait abbreviations: SMD—Snow Mold Damage; WH—Winter Hardiness; GR—Growth Retardation; PH—Plant Height; GY—Grain Yield; SL—Spike Length; NSS—Spikelets per Spike; NGS—Grains per Spike; GWS—Grain Weight per Spike; HGW—Hundred-Grain Weight. Trait vectors are colored according to the legend shown in the figure.

On the biplot (Figure 3), vectors for the disease-related traits (SMD, GR) pointed in the opposite direction to WH and yield vectors, confirming their antagonistic relationship (consistent with the correlation analysis in Figure 2). Because PC1 and PC2 together explain 48.2% of the total variance (Table S6), interpretations based on the biplot are limited to this portion of the variation.

Vectors for the same traits measured under different EIBs generally aligned with the corresponding NIB vectors within each trait group, indicating a consistent direction of association among traits across backgrounds. This reflects the general covariance structure among traits rather than accession-level rank consistency for individual traits such as SMD, which was only weakly correlated between backgrounds (Section 2.2; Table S5). Accessions showing the most favorable combination of resistance and productivity on the biplot (Grafinya, Sibirskaya 87, Kiprez, and VPK) clustered in the high-PC1/intermediate-PC2 quadrant, showing high projections onto the WH and GY vectors and low projections onto the SMD and GR vectors. In contrast, the three KWS hybrid cultivars (KWS Ravo, KWS Vendor, and KWS Akbator) occupied the lower end of the PC1 axis, reflecting their overall position on the composite resistance–productivity axis, which combines both higher disease severity and lower productivity under EIBs (Figure 3). The separation of these two groups along PC1 provides a visual framework for interpreting the subsequent Multi-trait Genotype–Ideotype Distance Index (MGIDI)-based multi-trait ranking.

### 2.5. Ranking of Winter Rye Accessions by the Multi-Trait Genotype–Ideotype Distance Index (MGIDI)

**Figure 4:**
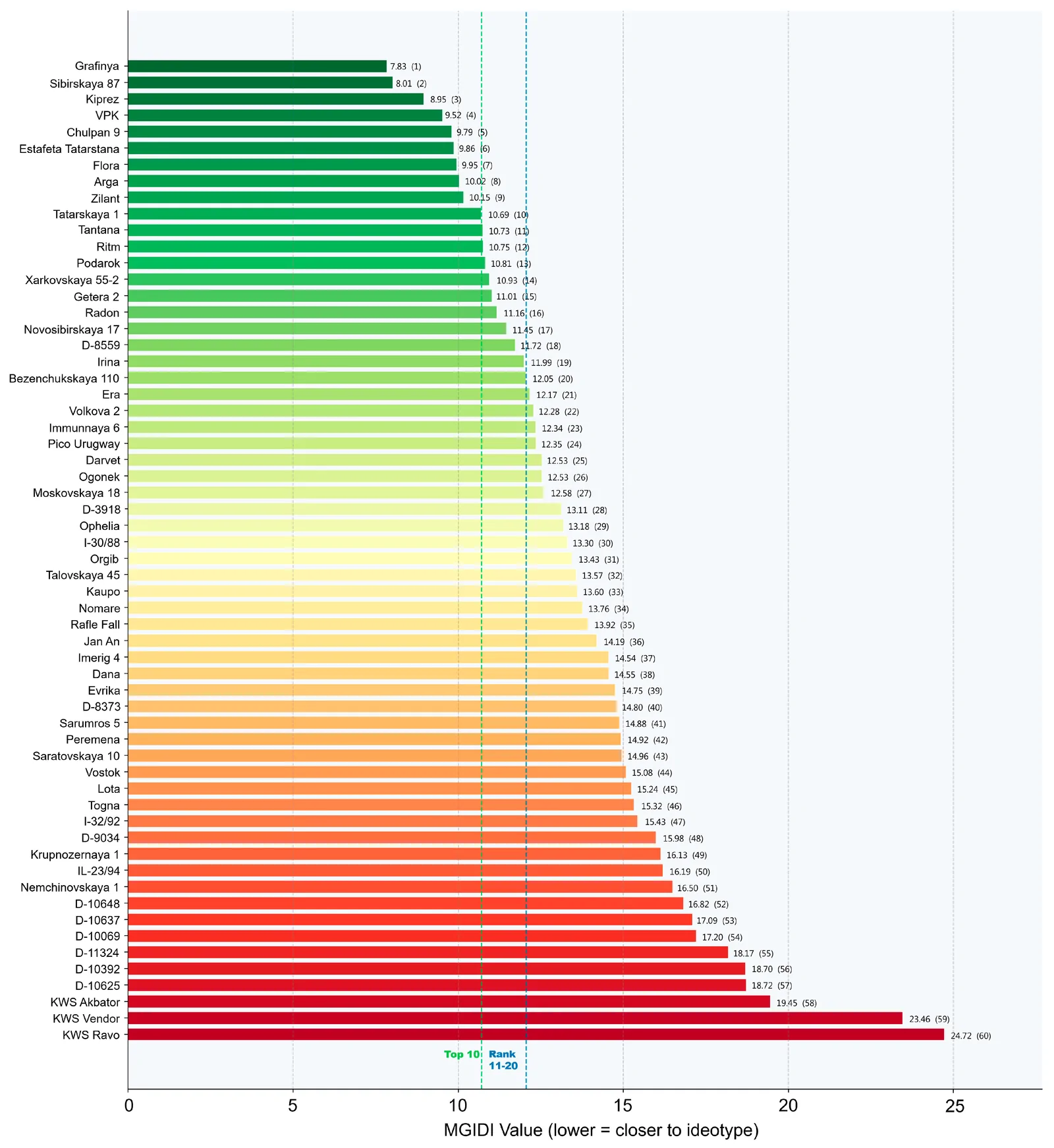
Multi-trait genotype–ideotype distance index (MGIDI) ranking of 60 winter rye accessions based on all evaluated traits across four infectious backgrounds. Lower MGIDI values indicate closer proximity to the ideotype and better overall performance. Ranks are shown in brackets. The green dashed line marks the top 10 performers, while the blue dashed line separates ranks 11–20. Column colors correspond to the MGIDI value: green—lowest, red—highest.

The resistance–productivity gradient visualized along PC1 of the biplot (Figure 3) was formalized into a single integrative criterion using the MGIDI (Figure 4). This index quantifies, in the eight-factor space retaining 81.2% of total variance, the Euclidean distance of each accession from a theoretically optimal ideotype defined by the best observed value of each trait across the 60 accessions. A lower MGIDI value therefore indicates superior simultaneous performance across all measured traits, formalizing the multivariate resistance–productivity gradient captured across all eight retained components, of which PC1 represents the dominant axis, into a single ranking criterion.

MGIDI values ranged ~3.2-fold, from 7.83 (Grafinya) to 24.72 (KWS Ravo), with a mean of 13.66 (Figure 4). The distribution was largely continuous, except for the two worst-ranked accessions in the upper tail (see below), consistent with the quantitative nature of the evaluated traits and the continuous distribution of accession scores along PC1.

The top-ranked accession, Grafinya (MGIDI = 7.83), combined the lowest SMD under NIB (2.00 points) with above-average WH (75.0%) and high GY (446.3 g m^−2^). Sibirskaya 87 (rank 2; MGIDI = 8.01) showed the highest WH under NIB among the top performers (81.2%) and the second-highest GY under NIB among the five top-ranked accessions (498.3 g m^−2^), despite a moderately elevated SMD (4.50 points). Ranks 3–5—Kiprez (MGIDI = 8.95), VPK (MGIDI = 9.52), and Chulpan 9 (MGIDI = 9.79)—combined low SMD (2.50–3.00 points) with high WH (75.0–78.8%) and GY (456.0–505.8 g m^−2^), forming a group of top performers.

In contrast, the three lowest-ranked accessions—KWS Akbator (rank 58; MGIDI = 19.45), KWS Vendor (rank 59; MGIDI = 23.46), and KWS Ravo (rank 60; MGIDI = 24.72)—showed the highest SMD values (5.50–6.25 points) and below-average WH under NIB. Applying the 1.5 × interquartile range rule to the distribution of MGIDI values (Q1 = 11.13, Q3 = 15.26, IQR = 4.13; upper fence = 21.46) identified KWS Vendor (MGIDI = 23.46) and KWS Ravo (MGIDI = 24.72) as statistical outliers, both exceeding the upper fence by a substantial margin.

**Figure 5:**
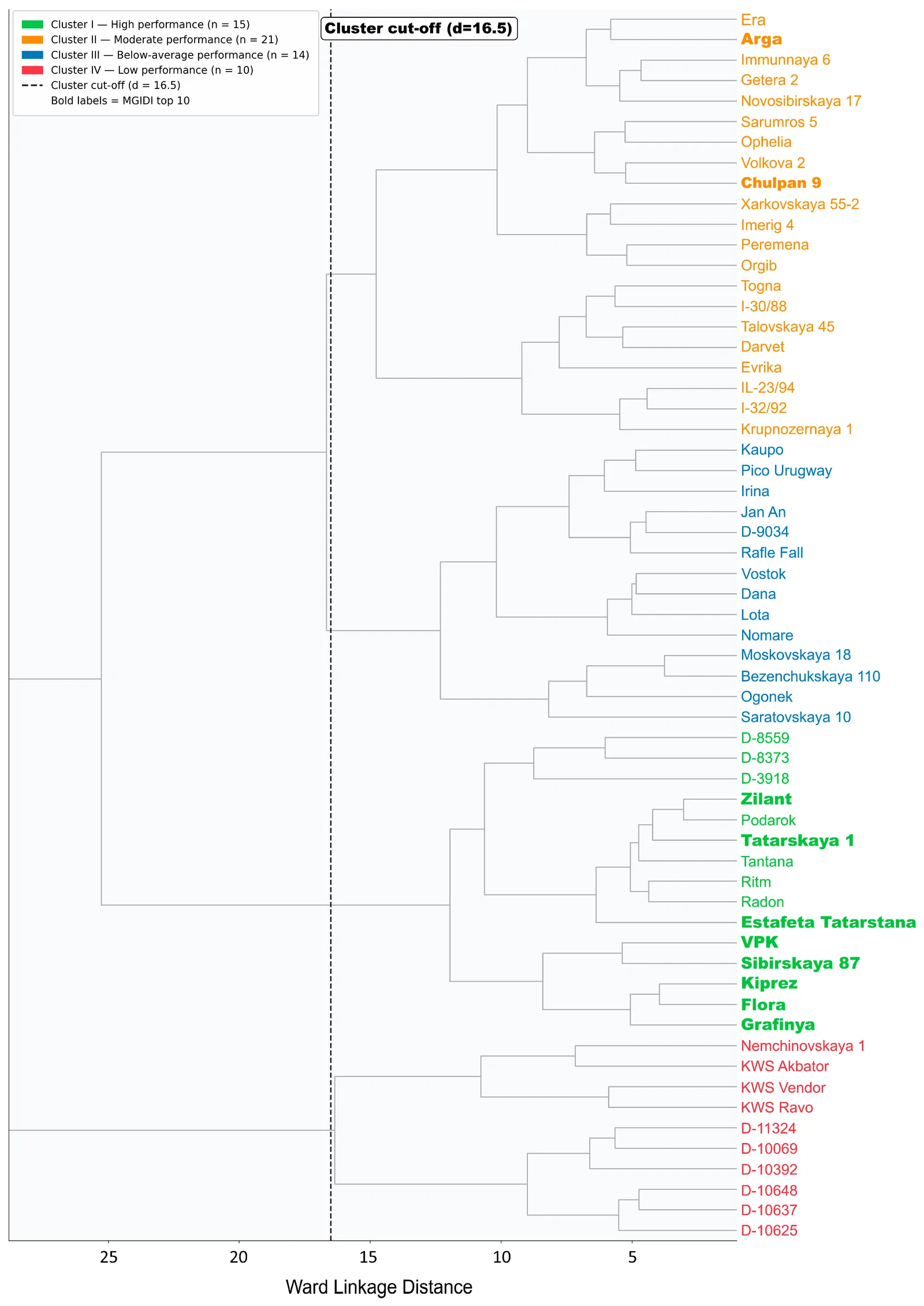
Hierarchical cluster dendrogram (Ward’s linkage method) of 60 winter rye accessions grown during the 2023–2024 and 2024–2025 growing seasons across four infectious backgrounds. The analysis was based on 40 standardized variables derived from 10 traits under four infectious backgrounds. Leaf label colors denote cluster membership: green = Cluster I (high performance, *n* = 15); orange = Cluster II (moderate performance, *n* = 21); blue = Cluster III (below-average performance, *n* = 14); red = Cluster IV (low performance, *n* = 10). Bold labels indicate the top 10 accessions according to MGIDI ranking.

Around ranks 10–12, an empirically chosen selection threshold of MGIDI < 11.0 is proposed as a practical cut-off for selecting candidate accessions for further breeding work (Figure 4), without implying a discrete discontinuity in the underlying distribution. Eight of the ten accessions below this threshold were assigned to Cluster I in the hierarchical cluster analysis (Section 2.6), although the clustering does not validate the exact threshold value (Figure 5).

To evaluate the reproducibility of accession rankings between the two growing seasons, which differed markedly in overwintering conditions (Table 3), accession × year interactions (Section 2.2; Table S3) and year-specific MGIDI rankings were additionally examined. The accession × year interaction was significant (*p* < 0.05) for six of the ten traits, including SMD and GY, but not for WH, SL, NGS, or GWS. Year-to-year Spearman rank correlations for individual traits ranged from non-significant for SMD (ρ = −0.26 to 0.18 across infectious backgrounds) to moderate and significant for WH and GY (ρ = 0.40–0.56 under NIB and most EIBs). The two seasonal MGIDI rankings were positively correlated (ρ = 0.54, *p* < 0.0001), although only four of the ten highest-ranked accessions (Bezenchukskaya 110, Chulpan 9, Ogonek, and Sibirskaya 87) were common to both seasons. Among these, only Chulpan 9 and Sibirskaya 87 also remained within the top ten of the pooled two-year ranking.

### 2.6. Hierarchical Cluster Analysis

To complement the MGIDI-based ranking, hierarchical cluster analysis was conducted using Ward’s minimum variance method. The resulting dendrogram (Figure 5) separated the accessions into four phenotypically distinct clusters based on their combined patterns of snow mold resistance, winter survival, and productivity.

Cluster I (*n* = 15; green labels in Figure 5) represented the most favorable group and showed the closest agreement with the highest MGIDI rankings. It included the majority of the top-performing accessions (Grafinya, Sibirskaya 87, Kiprez, VPK, Estafeta Tatarstana, Flora, Zilant, and Tatarskaya 1) together with several accessions that occupied intermediate MGIDI positions (Tantana, Ritm, Podarok, Radon, D-8559, D-3918, and D-8373). This cluster combined a low mean SMD under NIB (3.32; comparable to that of Cluster II, 3.27) with the highest WH (78.7%) and the highest GY (465.1 g m^−2^). The presence of several mid-ranked accessions indicates that they closely resemble the elite accessions across the complete 40-trait dataset, while differences in yield-related traits increase their overall MGIDI scores. For example, D-3918 (rank 28) and D-8373 (rank 40) exhibited low SMD and high WH comparable to the leading accessions but were penalized by lower productivity.

Cluster II (*n* = 21; orange labels) was the largest cluster, spanning a broad range of MGIDI ranks (5–50), indicating substantial diversity in overall multi-trait performance among its members. It included accessions with favorable snow mold resistance under NIB but lower productivity (e.g., Chulpan 9 and Arga), as well as numerous intermediate accessions (Xarkovskaya 55-2, Getera 2, Novosibirskaya 17, Era, Volkhova 2, and others). Notably, Chulpan 9 ranked among the top five accessions by MGIDI (rank 5) yet did not fall into the elite Cluster I group. This illustrates that MGIDI and hierarchical clustering can diverge: MGIDI ranks accessions by their overall Euclidean distance from the ideotype, whereas Ward’s clustering groups accessions by the overall similarity in multivariate trait space, which can differ even between top-ranked accessions. Although the average SMD under NIB (3.27) for accessions from Cluster II was comparable to that of Cluster I, WH (73.8%) and GY (402.4 g m^−2^) were substantially lower. These results indicate that resistance alone was insufficient to achieve high MGIDI rankings when accompanied by inferior agronomic performance.

Cluster III (*n* = 14; blue labels) grouped accessions with intermediate snow mold resistance (mean SMD under NIB = 4.05), the lowest WH among all clusters (69.8%), and reduced GY (374.6 g m^−2^). MGIDI rankings ranged from 19 (Irina) to 48 (D-9034). Most accessions in this cluster were characterized by moderate to poor snow mold resistance, including Moskovskaya 18, Ogonek, Nomare, Rafle Fall, Jan An, Dana, Saratovskaya 10, Vostok, Lota, Pico Urugway, Kaupo, and Bezenchukskaya 110. The relatively low WH likely contributed to reduced performance across several yield-related traits, resulting in less favorable MGIDI values.

Cluster IV (*n* = 10; red labels) was the most distinct group, joining the remaining clusters at the maximum Ward distance. It comprised exclusively the lowest-ranked accessions (MGIDI ranks 51–60), including the three KWS hybrid cultivars (KWS Akbator, KWS Vendor, and KWS Ravo), the Russian hybrid cultivar Nemchinovskaya 1, and six domestic accessions (D-10648, D-10637, D-10069, D-11324, D-10392, and D-10625). This cluster displayed the highest mean SMD under NIB (4.60), whereas its WH (74.6%) and GY (376.6 g m^−2^) were comparable to those of Cluster III. Accordingly, its unfavorable MGIDI ranking reflects the overall multivariate performance of these accessions across the full set of evaluated traits and infectious backgrounds rather than any single characteristic.

## 3. Discussion

In the present study, a diverse set of winter rye accessions was screened for resistance to overwintering stress that is strongly associated with snow mold disease. The goal was to identify accessions combining favorable levels of disease resistance, winter survival, regenerative capacity, and grain yield that could serve as parental material for broadening the genetic base of high-yielding cultivars adapted to regions with prolonged and persistent snow cover. This task is inherently challenging because “snow mold” is to some extent an umbrella term that encompasses diseases caused by phylogenetically distant fungi with distinct modes of interaction with the host, while disease development is strongly modulated by year-specific weather conditions [37,38]. Consequently, objective evaluation of germplasm for this trait is difficult. To address these challenges, we evaluated the accessions over two years under four infectious backgrounds, including three EIBs created with the major snow mold pathogens *M. nivale* (EIB-1), *T. incarnata* (EIB-2), and *T. ishikariensis* (EIB-3). Simultaneous evaluation of resistance to *M. nivale*, *T. incarnata*, and *T. ishikariensis* under standardized field conditions has not previously been reported for winter rye. This approach makes it possible to impose high disease pressure in backgrounds enriched with different pathogens instead of relying on the unpredictable natural infectious background of a given year. Previous multi-pathogen studies on snow mold resistance are very scarce and were conducted on winter wheat and winter barley [39,40]; rye germplasm has not been evaluated against a broad spectrum of snow mold pathogens. The experimental design employed allowed us to compare accession performance under a natural infectious background and under three backgrounds, each experimentally enriched with a different major snow mold pathogen, and thereby to assess the extent to which ranking of accessions for resistance to the snow mold complex as a whole is associated with the response under each enriched background individually. As detailed below, this comparison should be interpreted with caution, since pathogen enrichment was not the only factor distinguishing the EIB nurseries from NIB.

It should be acknowledged that the present design does not allow the effect of pathogen species to be separated cleanly from nursery and microenvironmental effects: the NIB and EIB nurseries were located ~1800 m apart, with the EIBs additionally sheltered by windbreaks and positioned outside the crop rotation, and randomization was performed within, rather than across, nurseries. Consequently, differences between NIB and the EIBs—and, to a lesser extent, among EIB-1–EIB-3 despite the 4-m buffer strips—may reflect location and snow microenvironment as well as pathogen enrichment; we also did not perform post-inoculation verification of pathogen identity or abundance (e.g., reisolation or qPCR), and the inoculum application rate (200 g m^−2^ for each pathogen) was calibrated to differentiate genotypes under each pathogen individually rather than to equalize viable inoculum density across species produced under different incubation regimes. For these reasons, we describe EIB-1–EIB-3 as backgrounds experimentally enriched with the respective pathogen rather than pure single-pathogen environments and interpret the observed differences among EIB-1–EIB-3 as indicative of differential host responses under pathogen-enriched infectious backgrounds rather than definitive evidence of pathogen-specific resistance.

Under NIB in the Volga region, where snow mold pressure is regularly high and, in some years, reaches epiphytotic levels, mean SMD was moderate (3.69 ± 0.87 points) with relatively high phenotypic variability (CV = 23.5%), reflecting differences in accession resistance. Enrichment of the infectious background in all three experimental treatments markedly and significantly increased disease severity (to 7.34 ± 0.60 under EIB-1, 6.54 ± 0.57 under EIB-2, and 7.41 ± 0.64 under EIB-3), corresponding to increases of 2.9–3.7 points relative to NIB. These results emphasize the importance of testing under epiphytotic conditions (under EIBs), as they allow evaluation of accession responses under high disease pressure, which is of particular relevance for breeding. While phenotypic variability of SMD decreased under the EIBs relative to NIB, variability in GY and WH increased. This indicates that effective ranking of accessions for breeding purposes requires consideration not only of disease severity itself but also of regenerative capacity and the ability to maintain yield under high disease pressure. This is the first study to simultaneously characterize the response of winter rye accessions under field backgrounds enriched with each of the three major snow mold pathogens.

The more severe SMD observed under the EIBs compared to NIB was accompanied by a pronounced decline in winter hardiness, confirming the close relationship between disease resistance and successful overwintering. None of the accessions were classified as resistant under the experimental conditions used, indicating the absence of complete resistance in the evaluated germplasm. This finding is consistent with the established view that snow mold resistance is a quantitative trait: it can reduce disease severity but is insufficient to prevent symptom development under strong infectious pressure [12,13].

Relative (quantitative) resistance revealed among the studied accessions was not universal across infectious backgrounds. No accession ranked among the most resistant under all four backgrounds, or even under three of them. Moreover, pairwise Spearman rank correlations of SMD between backgrounds were mostly weak or non-significant, and the accession × background interaction for SMD was significant (Table S3). Although these results do not by themselves demonstrate different resistance mechanisms, they indicate that resistance in the studied winter rye germplasm is heterogeneous and background-dependent, and possibly partly pathogen-specific. This pattern appears biologically plausible given the large phylogenetic distance between *Microdochium* (Ascomycota) and *Typhula* (Basidiomycota) and the correspondingly different plant–pathogen interaction mechanisms. Published evidence on this point is contradictory. Early work on winter wheat reported correlated resistance responses to *M. nivale*, *T. incarnata*, and *T. ishikariensis* and a lack of strong pathogenic specialization [40]. Other studies emphasize a more complex, partially independent nature of resistance components across snow mold pathogens [18,41,42]. Several accessions in the presented panel ranked among the leaders under both the NIB and one EIB (Kiprez and Ritm under NIB and EIB-1; D-3918 and Getera 2 under NIB and EIB-2; and Bezenchukskaya 110 under NIB and EIB-3). Others performed best under only one pathogen-enriched background: Ritm and Estafeta Tatarstana against *M. nivale*; Vostok and D-10392 against *T. ishikariensis*; and D-3918 against *T. incarnata*. These results suggest that different accessions may carry complementary resistance components that could be combined through crossing to improve adaptation to diverse pathogen complexes.

This study revealed that GY showed a stronger association with regenerative capacity than with initial disease severity: in a multivariable model including SMD, GR, WH, background, and year, GR retained a significant independent negative association with GY in every background (standardized β = −0.20 to −0.49, *p* < 0.001), whereas the independent association of SMD with GY was weak and mostly non-significant once GR and WH were accounted for (β = −0.02 to +0.07). This indicates that the agronomic consequences of snow mold infection are better explained by the capacity of surviving plants to resume vegetative growth than by the initial level of symptoms recorded immediately after snowmelt. Accessions with similar disease scores can therefore differ substantially in productivity because of differences in regenerative potential. These observations support an integrated breeding strategy that combines testing under multiple infectious backgrounds—because selection on a single EIB risks identifying genotypes with narrow, pathogen-specific resistance that may prove inadequate under a different pathogen complex—and evaluation across a set of breeding-relevant traits rather than disease severity alone. This further justifies the multi-trait selection framework applied in the present study.

Snow mold stress differentially affected individual yield components. SL and the NSS remained relatively stable across infectious backgrounds, whereas NGS and GWS declined markedly. This pattern is consistent with snow mold affecting grain-number-related traits more strongly than traits determined earlier in spike development, although direct floret-level effects were not assessed in this study. Consequently, NGS may represent a more sensitive indicator of tolerance to snow mold than GWS when phenotyping resources are limited. PH exhibited a similar trend, with the greatest reduction observed under EIB-3. Its positive association with winter hardiness and negative association with GR indicates that taller accessions generally combined better overwintering with more complete post-winter recovery; thus, no survival–recovery trade-off was evident.

The continuous distribution of MGIDI values was consistent with the quantitative nature of snow mold resistance inferred from the phenotypic data. This is consistent with, though does not by itself demonstrate, a polygenic basis for snow mold resistance involving multiple loci that limit disease development, enhance overwintering survival, and promote recovery after stress [29,30,43]; the presence or absence of major-effect loci would require genetic mapping to confirm. In this context, MGIDI provided an effective framework for simultaneous selection based on disease resistance, WH, regenerative capacity, and GY, as the index accounts for the correlations among traits and identifies accessions with balanced combinations of these attributes. The highest-ranked accessions (Grafinya, Sibirskaya 87, Kiprez, VPK, Chulpan 9, Estafeta Tatarstana, Flora, Arga, Zilant, and Tatarskaya 1) combined favorable resistance profiles with high mean multi-trait performance across the two growing seasons under snow mold pressure and represent valuable parental sources for breeding.

PCA further supported the multi-trait nature of snow mold resistance. The primary axis represented a resistance–productivity gradient, whereas the second axis mainly reflected variation in plant architecture and grain size, indicating that these traits vary partly independently of disease resistance. Notably, the three commercial KWS hybrid cultivars consistently occupied the lowest MGIDI ranks and the low-PC1 pole of the biplot, reflecting both high SMD and reduced productivity under EIBs. As these hybrids were not specifically selected for resistance to the snow mold pathogen complex, their comparatively poor performance under the severe experimental conditions most likely reflects limited adaptation to this particular disease pressure rather than generally inferior agronomic potential. These hybrids were developed in Western Europe for environments with short and unstable snow cover, where snow mold is not a primary selection target. This finding emphasizes the importance of evaluating breeding material under region-specific pathogen conditions.

Hierarchical cluster analysis largely supported the MGIDI-based ranking. The cluster of the highest-performing accessions (Cluster I) contained a substantial proportion of the top MGIDI accessions (Grafinya, Kiprez, VPK, and Estafeta Tatarstana) that combined below-average SMD under NIB, above-average WH, and the highest GY. Among accessions of Cluster I, two distinct phenotypic strategies were apparent. The first, a more resistance-balanced strategy, was exemplified by Grafinya and Kiprez, which combined low SMD, moderate GR, and good yield components. The second, a more yield-oriented strategy, was represented by Sibirskaya 87, VPK, Zilant, and Tatarskaya 1, in which high WH and GY compensated for moderately higher SMD. Both strategies produced similar MGIDI values, demonstrating that the index successfully integrates alternative, mutually compensating pathways to overall agronomic value.

Although the clustering broadly agreed with the MGIDI ranking, the two classifications did not correspond exactly. Minor discrepancies between MGIDI ranking and hierarchical clustering are expected, because the two methods optimize different criteria: MGIDI measures proximity to an ideotype, whereas clustering groups accessions according to overall phenotypic similarity. The four-cluster structure and the MGIDI rankings converged strongly at the extremes—Cluster I capturing the top performers and Cluster IV the poorest performers—while intermediate ranks were distributed across Clusters II–III. These results highlight the complementary nature of the two approaches. Together, they offer a robust framework for identifying promising sources of snow mold resistance while simultaneously considering adaptation and agronomic performance.

Collectively, the findings indicate that effective selection for snow mold resistance in winter rye should rely on a multi-trait approach rather than visual assessment of disease severity alone. Selection based exclusively on SMD scores may overlook productive accessions capable of maintaining yield through superior overwintering and post-winter recovery. Moreover, accessions showing only moderate resistance under one infectious background should not necessarily be excluded from breeding programs, as they may carry valuable alleles for pathogen-specific resistance or adaptive traits that can be combined through crossing. Integrating disease severity with winter hardiness, regenerative capacity, and grain yield provides a more comprehensive assessment of breeding value than any individual trait considered separately. Therefore, accessions identified by MGIDI and cluster analysis represent valuable parental material for the simultaneous improvement of resistance and agronomic performance.

The evaluated germplasm does not yet fully meet the requirements of regions with high snow mold pressure, underscoring the need to broaden the genetic base of breeding material with additional resistance sources. The present work represents an important step in the identification of parental forms for the development of cultivars that combine WH, resistance to multiple snow mold pathogens, and high yield potential.

## 4. Materials and Methods

### 4.1. Plant Material

This study evaluated 60 genetically diverse winter rye accessions (*S. cereale* L.). Germplasm was assembled from the collection of the N.I. Vavilov Institute of Plant Genetic Resources (VIR, St. Petersburg, Russia) and Tatar Scientific Research Institute of Agriculture (TatSRIA, Federal Research Center “Kazan Scientific Center of the Russian Academy of Sciences”, KSC). The set included the released cultivars, hybrids, advanced lines and landraces; cultivar Tantana was included as a local check. Sixty-six percent of the accessions were of Russian origin, with the remaining 34% originating from 10 countries: Belarus, Latvia, Germany, Uruguay, China, Canada, Ukraine, Bulgaria, Serbia, and Finland (listed in Table S8).

For non-hybrid accessions (e.g., open-pollinated varieties and landraces), seed stocks were renewed each year by cultivating individual accessions under isolation to maintain genetic purity. In contrast, the four commercial F1 hybrids (Nemchinovskaya 1 and three KWS hybrids listed in Table S8) were represented in each experimental season by freshly obtained certified commercial seed and were therefore not subject to regeneration.

Each accession was cultivated on 1.5 m^2^ plots under cotton isolation cabins during the flowering period. After manual harvesting, seed material was processed using a Wintersteiger LD 180 plot thresher (Wintersteiger, Ried im Innkreis, Austria).

### 4.2. Study Area and Climate

Field experiments were conducted at the TatSRIA experimental station (55.649° N, 49.308° E; Laishev District, Republic of Tatarstan, Russia) during two consecutive growing seasons: 2023–2024 and 2024–2025. The site has a moderately continental climate (mean January temperature −14.2 °C; mean July temperature +19.2 °C; annual precipitation − 610 mm). Stable snow cover typically establishes in November and persists until mid-April, with a mean depth of 30–35 cm. The frost-free period extends from late May to late September (approximately 120 frost-free days per year). Under long-term average climatic conditions, ground frost depth generally ranges from 60 to 92 cm, whereas maximum frost depths of 100–120 cm have been recorded during winters with limited snow cover. Spring thaw occurs in mid-April (Table S9).

Meteorological observations were recorded daily by the on-site weather station for the duration of both experimental seasons. The two seasons were characterized by differing levels of snow cover depth and duration. Overall, this offered a broad range of conditions, providing a useful basis for the assessment of resistance (Table 3).

### 4.3. Experimental Design

The set of 60 winter rye accessions was evaluated separately under four infectious backgrounds: natural infectious background (NIB) without pathogen supplementation and three experimental infectious backgrounds (EIBs) with *Microdochium nivale* (EIB-1), *Typhula incarnata* (EIB-2), and *Typhula ishikariensis* (EIB-3). The NIB and EIB nurseries were located 1800 m apart to prevent cross-contamination. The three EIB nurseries (EIB-1, EIB-2, and EIB-3) were separated from one another by 4-m buffer strips sown with winter camelina. EIB nurseries were positioned outside the crop rotation and sheltered on three sides by windbreaks to promote snow retention and uniform pathogen development.

Field trials in both seasons followed a randomized complete block design with two replicates per year. Each plot consisted of eight rows 2.5 m in length with 15 cm row spacing (plot area 3 m^2^), with a seeding density of 450 seeds m^−2^. Sowing was performed simultaneously on 29–30 August using identical agronomic practices across both NIB and EIB locations.

Soil at all sites consisted of gray forest podzolic soils of moderate fertility (organic matter 3.1%; pH 5.8; total nitrogen 112.0 mg kg^−1^; P_2_O_5_ 242 mg kg^−1^; K_2_O 56.5 mg kg^−1^). The preceding crop was bare fallow. No fungicides were applied. Pre-sowing fertilization comprised diammonium phosphate at 120 kg ha^−1^; in spring, 100 kg ha^−1^ ammonium nitrate (33.5% N) was applied at BBCH growth stage 21. Weeds were controlled with Agritox VK at a rate of 1.5 L ha^−1^.

### 4.4. Enrichment of Infectious Backgrounds

Enrichment of infectious backgrounds was carried out according to the protocol previously described [35]. For this purpose, strains previously genotyped and phenotyped as the most virulent from the collection of the Laboratory of Plant Infectious Diseases, KSC RAS [37,44] were selected: *M*. *nivale* (eight strains), *T. incarnata* (two strains), and *T. ishikariensis* (three strains). These strains originated from the Volga region and were isolated in 2019–2020 from snow mold-affected winter crops.

Fungi were grown under sterile conditions on potato–sucrose agar (PSA). Plug discs (8 mm in diameter) cut from colony margins were transferred into 3-L glass flasks containing half-filled autoclaved crushed spring barley grain. *M. nivale* strains were incubated for 16 days at 20 °C with regular substrate agitation. *Typhula* strains were incubated for six weeks at 5 °C with regular substrate agitation. Immediately before the colonized grain was applied to the experimental plots, grain inoculum from individual strains belonging to the same species was mixed in equal proportions (by weight) and then blended with sterile autoclaved barley grain in a 1:3 ratio (*v*/*v*) to increase volume while reducing inoculum density relative to the high aggressiveness of the selected strains.

Inoculum was hand-applied evenly between crop rows at 200 g m^−2^ (i.e., approximately 50 g m^−2^ of colonized grain) after plants reached the three-leaf stage (Zadoks growth stage 13). The application rate of 200 g m^−2^ of the inoculum mixture for each pathogen was established in prior work [35] as sufficient to produce reproducible disease pressure discriminating among genotypes under that pathogen. This rate was not calibrated to achieve equivalent viable inoculum density across the three pathogen species, which were produced under different incubation regimes; potential differences in effective inoculum pressure among EIBs are addressed as a limitation in the Discussion Section.

### 4.5. Disease Assessment and Trait Evaluation

Snow mold severity was assessed twice: immediately after snowmelt, to record plant damage (Snow Mold Damage score, SMD); and 20–25 days later (depending on weather conditions), to evaluate post-disease recovery (Growth Retardation score, GR). Both traits were scored on a 1–9 point scale (Table S10), where 1 indicates no visible damage or full recovery, and 9 indicates plant death or no regrowth. This two-stage assessment captures both the susceptibility of the accession at the peak of disease development and its capacity for regeneration after the disease peak, and enables indirect estimation of tiller loss and the associated yield reduction.

For overall resistance classification, the accessions were assigned to categories based on their mean SMD score (mean of two replicates and two years) under each of the four infectious backgrounds separately: mean SMD ≤ 3.0—Resistant (R); 3.0 < SMD ≤ 5.0—Moderately resistant (MR); 5.0 < SMD ≤ 7.0—Moderately susceptible (MS); SMD > 7.0—Susceptible (S). GR was scored on the same 1–9 scale 20–25 days after snowmelt according to the extent of post-winter regrowth (proportion of regrowing plants, formation of new leaves, and condition of tillering shoots), from 1 = complete recovery to 9 = no regrowth (Table S10). Winter hardiness (WH, %) was determined as the percentage of plants that survived the winter, calculated by counting plants in autumn (before snow cover) and in spring after full snowmelt.

In addition to disease scores and WH, the following agronomic traits were recorded from the central harvest rows: grain yield (GY, g m^−2^), plant height (PH, cm), spike length (SL, cm), number of spikelets per spike (NSS), number of grains per spike (NGS), grain weight per spike (GWS, g), and hundred-grain weight (HGW, g). All ten traits were evaluated under all four infectious backgrounds (NIB, EIB-1, EIB-2, EIB-3), yielding 40 variables per accession. Grain yield and yield-component traits were sampled from the same central harvest rows. Prior to combine harvesting, 15 plants were removed from the central rows for spike-level trait assessment (SL, NSS, NGS, GWS, HGW); grain from these plants was threshed, weighed, and adjusted to 14% moisture content in the same manner as the combine-harvested grain. Grain yield (GY, g m^−2^) was calculated as the sum of the combine-harvested grain and the grain from the 15 sampled plants (both standardized to 14% moisture), divided by the gross plot harvest area (3 m^2^).

### 4.6. Statistical Analysis

Descriptive statistics (mean, SD, CV, minimum, maximum, and interquartile range) were calculated for all traits. Potential outliers were identified by the 1.5 × IQR criterion; no observations were removed, as no measurement or recording errors were found.

Differences among infectious backgrounds were tested on accession means with the Friedman test for related samples (accessions as blocks), followed by pairwise Wilcoxon signed-rank tests with Bonferroni correction.

Within each background, accession and year effects were tested by two-way ANOVA (Table S2). In addition, a combined ANOVA (Type III sums of squares) including accession, background, year, and the accession × background and accession × year interactions was fitted to the plot-level data (Table S3). Replicate (block) effects and the background × year interaction were not fitted as separate terms and were therefore pooled into the residual error; this makes the F-tests for the accession effect and the accession-related interactions conservative. Because NIB and the EIBs were located in separate nurseries, this model was used to test accession effects and accession-related interactions, whereas background comparisons relied on the Friedman test. Residuals showed only minor deviations from normality (Shapiro–Wilk W ≥ 0.988).

Associations among traits were evaluated using Spearman rank correlations (ρ) of accession means averaged across backgrounds (*n* = 60). Correlations of SMD between backgrounds were tested with Holm correction for multiple comparisons (Table S5), and agreement between seasons was assessed by Spearman correlations of year-specific accession means and year-specific MGIDI rankings. The associations of GY with SMD, GR, and WH were further examined by multiple linear regression on standardized variables, fitted to the pooled data (with background and year as factors) and separately for each background.

Multivariate analyses were based on accession means across replicates and years. PCA was applied to the 40 standardized trait × background variables, and components with eigenvalues ≥ 1 were retained. Hierarchical clustering was performed using Euclidean distances and Ward’s method; the number of clusters (k = 4) was chosen from the dendrogram.

Overall performance was assessed with the Multi-trait Genotype–Ideotype Distance Index (MGIDI) [45,46]. In a modification of the original procedure, which uses factor analysis with varimax rotation [46], MGIDI was computed as the Euclidean distance of each accession from the ideotype in the space of the eight retained principal components (81.2% of total variance; Table S6), with all variables weighted equally. The ideotype combined minimum SMD and GR with maximum values of all other traits (Table S11); its coordinate on each component was set to the most favorable observed accession score. Lower MGIDI values indicate closer proximity to the ideotype (Table S12). PH was maximized as a proxy for overwintering and post-winter regrowth, given its positive correlation with WH and negative correlation with GR (Figure 2); lodging risk was not assessed.

Within-background ANOVAs were performed in GraphPad Prism 9.5.1 (GraphPad Software, Boston, MA, USA); all other analyses were performed in Python 3.12 (statsmodels 0.14, scikit-learn 1.4, SciPy 1.13, scikit-posthocs 0.9.0, NumPy 1.26, Matplotlib 3.8).

The complete set of experimental results for SMD and all evaluated variables is provided in Table S13, and the results of the Friedman test and pairwise comparisons are in Table S14.

## 5. Conclusions

The present study advances an integrated approach to practical breeding by combining multi-condition testing under natural and experimentally enriched infectious conditions with multi-trait evaluation of each accession. This framework was applied to improve winter rye resistance to snow mold while maintaining agronomic performance under variable winter conditions.

Snow mold resistance in winter rye is a quantitative trait closely linked to winter hardiness, post-winter recovery, and grain yield. Experimental enrichment of the infectious background with highly virulent pathogens, particularly *T. ishikariensis*, produced stronger and more uniform disease pressure than natural infection. This increased phenotypic variation among accessions in winter hardiness and grain yield, whereas variation in SMD decreased, possibly reflecting a ceiling effect of the scoring scale; the EIBs thus enabled assessment of resistance under severe, breeding-relevant disease pressure.

Practical selection for snow mold resistance should not rely solely on visual assessment of disease severity after snowmelt. In the present study, grain yield under snow mold stress was more strongly associated with the capacity for post-winter regeneration than with the initial level of symptoms. Effective selection must therefore target a combination of traits that together determine plant productivity under prolonged winter stress, including winter hardiness, regenerative capacity, and the ability to form yield following disease development.

Accessions identified in this study are recommended as parental material for breeding programs aimed at improving resistance to the snow mold complex and maintaining favorable agronomic performance under winter stress. These include both accessions with the most balanced multi-trait performance across the two evaluated seasons (Grafinya, Kiprez, Chulpan 9, VPK, and Estafeta Tatarstana) and those displaying superior performance under particular pathogen-enriched backgrounds (Ritm and Estafeta Tatarstana under EIB-1, enriched with *M. nivale*; D-3918 under EIB-2, enriched with *T. incarnata*; Vostok and D-10392 under EIB-3, enriched with *T. ishikariensis*). These accessions represent complementary breeding resources, providing either broad multi-trait adaptation or distinct response profiles across pathogen-enriched infectious backgrounds.

## Figures and Tables

**Table 1:** Distribution of 60 winter rye accessions by snow mold damage (SMD) categories. Accessions were grown during the 2023–2024 and 2024–2025 growing seasons across four infectious backgrounds: natural infectious background (NIB), or experimentally enriched infectious backgrounds (EIBs) with *Microdochium nivale* (EIB-1), *Typhula incarnata* (EIB-2), or *Typhula ishikariensis* (EIB-3). Data are shown as the number of accessions (percentage of the total). Categories are defined according to SMD as follows: mean SMD ≤ 3.0—Resistant (R); 3.0 < SMD ≤ 5.0—Moderately resistant (MR); 5.0 < SMD ≤ 7.0—Moderately susceptible (MS); SMD > 7.0—Susceptible (S).

Background	NIB	*M. nivale* (EIB-1)	*T. incarnata* (EIB-2)	*T. ishikariensis* (EIB-3)
Resistant (%)	15 (25.0)	0 (0.0)	0 (0.0)	0 (0.0)
Moderately resistant (%)	38 (63.3)	0 (0.0)	1 (1.7)	0 (0.0)
Moderately susceptible (%)	7 (11.7)	20 (33.3)	52 (86.7)	22 (36.7)
Susceptible (%)	0 (0.0)	40 (66.7)	7 (11.7)	38 (63.3)

**Table 2:** Ranking of accessions with the lowest snow mold damage (SMD) under each of the four infectious backgrounds (SMD values (points) are given in brackets). NIB—natural infectious background; EIB-1–EIB-3—experimentally enriched infectious backgrounds with *Microdochium nivale* (EIB-1), *Typhula incarnata* (EIB-2), or *Typhula ishikariensis* (EIB-3).

Rank	NIB	*M. nivale* (EIB-1)	*T. incarnata* (EIB-2)	*T. ishikariensis* (EIB-3)
1	Grafinya (2.0)	Estafeta Tatarstana, Ritm (6.0)	D-3918 (5.0)	Vostok, D-10392 (6.0)
2	Chulpan 9, D-3918, Kiprez, I-30/88 (2.5)	Nemchinovskaya 1, Kiprez (6.3)	Tatarskaya 1 (5.5)	Bezenchukskaya 110, Irina, Lota (6.3)
3	Bezenchukskaya 110, Getera 2, Volkhova 2, Evrika, Ritm (2.8)	Tatarskaya 1, Evrika, Grafinya, I-30/88, Darvet (6.5–6.8)	Getera 2, Arga, Irina, Ophelia (5.8)	D-10069, Getera 2, Nomare (6.5)

**Table 3:** Seasonal meteorological parameters and snow mold damage at the experimental site (2023–2024 and 2024–2025). NIB—natural infectious background; snow mold damage values are means ± SD across all 60 accessions.

Indicator	2023–2024	2024–2025
Duration of cold period, days	128	102
Snow cover duration, days	136	98
Number of thaw days	34	29
Maximum snow cover height, cm	95	35
Snow cover height as of 31 March, cm	30	0
Maximum soil freezing depth, cm	7	15
Average air temperature, cold period (Nov–Mar), °C (long-term norm: −7.4 °C)	−7.4	−3.2
Total precipitation, cold period (Nov–Mar), mm	310	155
Snow mold damage, NIB (mean ± SD, points)	4.77 ± 1.35	2.61 ± 0.73

## Data Availability

The original contributions presented in this study are included in the article/Supplementary Materials. Further inquiries can be directed to the corresponding authors.

## Supplementary Materials

The following supporting information can be downloaded at: https://www.mdpi.com/article/10.3390/plants15213375/s1, Table S1: Descriptive statistics (mean, SD, CV, range, and interquartile range) for ten traits in 60 winter rye (*S. cereale*) accessions under natural infectious background (NIB) and three experimentally enriched infectious backgrounds (EIB-1–EIB-3); Table S2: Two-way ANOVA of accession effects on agronomic and disease-related traits in 60 winter rye (*S. cereale*) accessions evaluated under natural infectious background (NIB) and three experimentally enriched infectious backgrounds (EIB-1–EIB-3); Table S3: Combined analysis of variance (accession, background × year, replicate within background × year, accession × background, and accession × year) for ten agronomic and disease-related traits in 60 winter rye (*S. cereale*) accessions, based on plot-level data (*n* = 960 observations per trait; 60 accessions × 4 backgrounds × 2 years × 2 replicates); Table S4: Significant Spearman rank correlations (ρ) among trait means of 60 winter rye (*S. cereale*) accessions, averaged across four infectious backgrounds (NIB, EIB-1, EIB-2 and EIB-3); Table S5: Pairwise Spearman rank correlations of snow mold damage (SMD) among the four infectious backgrounds (NIB, EIB-1, EIB-2 and EIB-3) for 60 winter rye (*S. cereale*) accessions, with 95% confidence intervals and Holm-adjusted *p*-values; Table S6: Eigenvalues and variance explained by the eight principal components (PC) with eigenvalue ≥ 1, retained from principal component analysis of 60 winter rye (*S. cereale*) accessions; Table S7: Complete matrix of principal component loadings (40 trait × background variables on the eight retained components) underlying the principal component analysis of 60 winter rye (*S. cereale*) accessions; Table S8: List of the 60 winter rye (*S. cereale*) accessions evaluated in this study, including accession code, accession name, seed source, country of origin, and accession type; Table S9: Long-term average winter climatic parameters at the study site (Laishev District, Republic of Tatarstan, Russia); Table S10: Nine-point scale (1–9) used to score snow mold damage (SMD) immediately after snowmelt and post-winter growth retardation (GR) 20–25 days later in winter rye (*S. cereale*) based on crop damage, leaf symptoms, and tillering-shoot symptoms; Table S11: Ideotype definition used to calculate the Multi-Trait Genotype–Ideotype Distance Index (MGIDI) for 60 winter rye (*S. cereale*) accessions; Table S12: Multi-trait genotype–ideotype distance index (MGIDI) values and final ranks for 60 winter rye (*S. cereale*) accessions; Table S13: Plot/plant-level values of ten phenotypic traits for 60 winter rye (*S. cereale*) accessions evaluated in two growing seasons (2023–2024 and 2024–2025) and two field replicates under four infectious backgrounds (NIB, EIB-1, EIB-2 and EIB-3); Table S14: Friedman test for differences among the four infectious backgrounds and pairwise Wilcoxon signed-rank comparisons (Bonferroni-corrected) for ten traits in 60 winter rye (*S. cereale*) accessions.

## Abbreviations

The following abbreviations are used in this manuscript:

MGIDI	Multi-trait Genotype–Ideotype Distance Index
NIB	Natural infectious background
EIB	Experimentally enriched infectious background
EIB-1	Experimentally enriched infectious background 1 (with *M. nivale*)
EIB-2	Experimentally enriched infectious background 2 (with *T. incarnata*)
EIB-3	Experimentally enriched infectious background 3 (with *T. ishikariensis*)
PSA	Potato–sucrose agar
SMD	Snow mold damage
R	Resistant
MR	Moderately resistant
MS	Moderately susceptible
S	Susceptible
GR	Growth retardation
WH	Winter hardiness
GY	Grain yield
PH	Plant height
SL	Spike length
NSS	Number of spikelets per spike
NGS	Number of grains per spike
GWS	Grain weight per spike
HGW	Hundred-grain weight
SD	Standard deviation
CV	Coefficient of variation
IQR	Interquartile range
PCA	Principal component analysis
