# Supplementary material for: From Snow Mold Severity to Yield Performance: Identifying Promising Winter Rye Accessions Through Multi-Pathogen and Multi-Trait Assessment
Source: Plants (Basel). 2026 Nov 1;15(21):3375. doi: 10.3390/plants15213375 (PMC13765948; doi:10.3390/plants15213375)
Supplement: Supplementary file 1 [file plants-15-03375-s001.zip › Tables S1-S12, S14.pdf]

Table S1. Descriptive statistics (mean, SD, CV, range, and interquartile range) for ten traits in 60 winter rye (*Secale cereale*) accessions under natural infectious background (NIB) and three experimentally enriched infectious backgrounds (EIB-1–EIB-3). Values are accession means pooled across two replicates and two growing seasons (2023-2024 and 2024-2025).

Trait	Background	Mean	SD	CV (%)	Min	Max	Q1 – Q3
SMD	NIB	3.69	0.87	23.5	2.00	6.25	3.19 – 4.25
SMD	EIB-1	7.34	0.60	8.1	6.00	9.00	7.00 – 7.75
SMD	EIB-2	6.54	0.57	8.7	5.00	7.75	6.25 – 6.75
SMD	EIB-3	7.41	0.64	8.7	6.00	8.50	7.00 – 8.00
WH	NIB	74.21	5.80	7.8	61.25	85.00	70.00 – 78.75
WH	EIB-1	36.96	8.15	22.0	23.75	57.50	28.75 – 42.50
WH	EIB-2	43.27	8.25	19.1	22.50	56.25	37.50 – 49.06
WH	EIB-3	31.60	8.09	25.6	17.50	48.75	25.00 – 37.50
GR	NIB	3.23	0.65	20.1	2.00	4.50	2.75 – 3.75
GR	EIB-1	5.80	0.80	13.7	4.25	7.50	5.25 – 6.25
GR	EIB-2	4.89	0.94	19.2	3.50	7.50	4.25 – 5.25
GR	EIB-3	6.19	0.62	10.0	4.75	7.50	5.75 – 6.50
PH (cm)	NIB	128.62	17.45	13.6	102.50	167.50	115.94 – 142.81
PH (cm)	EIB-1	115.88	14.12	12.2	82.50	151.25	106.25 – 123.12
PH (cm)	EIB-2	125.98	16.49	13.1	78.75	158.75	114.69 – 137.81
PH (cm)	EIB-3	109.85	15.07	13.7	82.50	141.25	98.75 – 121.56
GY (g/m²)	NIB	407.26	67.08	16.5	298.50	539.50	361.19 – 456.12
GY (g/m²)	EIB-1	144.31	42.84	29.7	56.50	226.25	108.75 – 173.50
GY (g/m²)	EIB-2	151.46	41.27	27.2	42.50	207.50	125.88 – 183.94
GY (g/m²)	EIB-3	85.53	27.59	32.3	40.00	153.00	63.88 – 102.69
SL (cm)	NIB	12.34	1.01	8.2	10.00	14.18	11.51 – 13.10
SL (cm)	EIB-1	11.44	0.92	8.1	9.05	13.75	10.84 – 12.06
SL (cm)	EIB-2	11.98	1.00	8.3	9.07	13.85	11.48 – 12.68
SL (cm)	EIB-3	11.97	0.93	7.8	9.05	13.78	11.66 – 12.55
NSS	NIB	36.30	2.05	5.6	32.10	40.90	35.24 – 37.59

Trait	Background	Mean	SD	CV (%)	Min	Max	Q1 – Q3
NSS	EIB-1	33.93	1.95	5.7	29.10	38.42	32.79 – 35.32
NSS	EIB-2	35.43	2.14	6.0	29.60	38.92	34.37 – 36.90
NSS	EIB-3	35.06	2.26	6.4	27.22	38.78	34.20 – 36.64
NGS	NIB	57.61	6.36	11.0	36.72	72.18	54.11 – 60.64
NGS	EIB-1	44.05	4.55	10.3	31.82	54.55	40.68 – 46.74
NGS	EIB-2	48.65	6.06	12.5	32.58	61.98	44.06 – 52.65
NGS	EIB-3	46.83	6.85	14.6	30.28	60.35	42.57 – 51.91
GWS (g)	NIB	2.01	0.35	17.4	1.03	2.70	1.78 – 2.23
GWS (g)	EIB-1	1.49	0.23	15.4	0.98	2.03	1.28 – 1.67
GWS (g)	EIB-2	1.64	0.28	17.2	1.01	2.17	1.45 – 1.84
GWS (g)	EIB-3	1.46	0.33	22.5	0.72	2.00	1.22 – 1.76
HGW (g)	NIB	3.57	0.43	12.1	2.62	4.78	3.27 – 3.90
HGW (g)	EIB-1	3.24	0.42	12.8	1.87	4.49	3.03 – 3.53
HGW (g)	EIB-2	3.44	0.37	10.7	2.69	4.36	3.18 – 3.67
HGW (g)	EIB-3	3.20	0.39	12.1	2.31	3.97	2.96 – 3.46

Table S2. Two-way ANOVA of accession effects on agronomic and disease-related traits in 60 winter rye (*Secale cereale*) accessions evaluated under natural infectious background (NIB) and three experimentally enriched infectious backgrounds (EIB-1–EIB-3). Analyses were performed separately for each background, with accession and year as fixed effects. Shown are F- and p-values for the accession main effect.

Trait	NIB (F)	EIB-1 (F)	EIB-2 (F)	EIB-3 (F)	NIB (p)	EIB-1 (p)	EIB-2 (p)	EIB-3 (p)
Grain weight per spike (GWS, g)	22.25	8.71	10.65	15.54	<0.0001 ****	<0.0001 ****	<0.0001 ****	<0.0001 ****
Grain yield per 1 m2 (GY, g)	8.56	5.74	9.28	9.34	<0.0001 ****	<0.0001 ****	<0.0001 ****	<0.0001 ****
Growth retardation (GR, points)	3.43	2.80	5.89	3.01	<0.0001 ****	<0.0001 ****	<0.0001 ****	<0.0001 ****
Hundred-grain weight (HGW, g)	22.49	17.76	16.71	20.46	<0.0001 ****	<0.0001 ****	<0.0001 ****	<0.0001 ****
Number of grains	17.53	7.57	11.22	17.47	<0.0001	<0.0001	<0.0001	<0.0001

per spike (NGS)					****	****	****	****
Number of spikelets per spike (NSS)	9.69	4.97	7.05	8.67	<0.0001 ****	<0.0001 ****	<0.0001 ****	<0.0001 ****
Plant height (PH, cm)	21.49	10.91	10.36	8.71	<0.0001 ****	<0.0001 ****	<0.0001 ****	<0.0001 ****
Snow mold damage (SMD, points)	2.72	2.92	1.83	2.77	<0.0001 ****	<0.0001 ****	0.0028 **	<0.0001 ****
Spike length (SL, cm)	9.34	4.90	6.85	6.49	<0.0001 ****	<0.0001 ****	<0.0001 ****	<0.0001 ****
Winter hardiness (WH, %)	3.28	5.82	6.23	6.40	<0.0001 ****	<0.0001 ****	<0.0001 ****	<0.0001 ****

Table S3. Combined analysis of variance for ten agronomic and disease-related traits in 60 winter rye (*Secale cereale*) accessions evaluated under four infectious backgrounds in two growing seasons, based on plot-level data (n = 960 observations per trait; 60 accessions × 4 backgrounds × 2 years × 2 replicates). Background × year and replicate (block) nested within background × year were included as separate terms; accession effects and accession-related interactions were tested against the residual mean square (df = 649). Background and year main effects are not shown because they are confounded with nursery and season; background comparisons are based on the Friedman test. SS% – percentage of the total sum of squares.

**Trait**	**Replicate (Background × Year) (df = 8)**	**Accession (df = 59)**	**Accession × Background (df = 177)**	**Accession × Year (df = 59)**
Snow mold damage (SMD)	F = 0.95; p = 0.477; SS% = 0.1	F = 3.07; p < 0.001; SS% = 3.3	F = 1.63; p < 0.001; SS% = 5.2	F = 2.42; p < 0.001; SS% = 2.6
Winter hardiness (WH)	F = 1.59; p = 0.124; SS% = 0.1	F = 8.90; p < 0.001; SS% = 5.2	F = 2.42; p < 0.001; SS% = 4.2	F = 3.14; p < 0.001; SS% = 1.8
Growth retardation (GR)	F = 1.84; p = 0.067; SS% = 0.4	F = 6.74; p < 0.001; SS% = 11.6	F = 2.02; p < 0.001; SS% = 10.5	F = 2.07; p < 0.001; SS% = 3.6
Plant height (PH)	F = 1.72; p = 0.090; SS% = 0.3	F = 27.02; p < 0.001; SS% = 39.0	F = 2.18; p < 0.001; SS% = 9.4	F = 5.47; p < 0.001; SS% = 7.9
Grain yield (GY)	F = 1.70; p = 0.096; SS% = 0.1	F = 12.07; p < 0.001; SS% = 4.7	F = 3.11; p < 0.001; SS% = 3.6	F = 6.19; p < 0.001; SS% = 2.4
Spike length (SL)	F = 46.04; p < 0.001; SS% = 8.8	F = 21.36; p < 0.001; SS% = 30.1	F = 2.78; p < 0.001; SS% = 11.8	F = 2.76; p < 0.001; SS% = 3.9
Number of spikelets per spike (NSS)	F = 34.72; p < 0.001; SS% = 5.9	F = 16.55; p < 0.001; SS% = 20.9	F = 3.01; p < 0.001; SS% = 11.4	F = 3.93; p < 0.001; SS% = 5.0
Number of grains per spike (NGS)	F = 22.50; p < 0.001; SS% = 1.7	F = 16.10; p < 0.001; SS% = 8.8	F = 4.16; p < 0.001; SS% = 6.8	F = 4.11; p < 0.001; SS% = 2.2
Grain weight per spike (GWS)	F = 28.23; p < 0.001; SS% = 1.9	F = 21.66; p < 0.001; SS% = 10.9	F = 5.06; p < 0.001; SS% = 7.7	F = 5.41; p < 0.001; SS% = 2.7
Hundred-grain weight (HGW)	F = 21.44; p < 0.001; SS% = 4.0	F = 17.34; p < 0.001; SS% = 23.6	F = 4.30; p < 0.001; SS% = 17.5	F = 4.03; p < 0.001; SS% = 5.5

Table S4. Significant Spearman rank correlations (ρ) among trait means of 60 winter rye (*Secale cereale*) accessions, averaged across four infectious backgrounds (NIB, EIB-1, EIB-2 and EIB-3). Only significant associations are shown (** p < 0.01; *** p < 0.001). The following 10 traits were assessed: Snow mold damage (SMD), Winter hardiness (WH), Growth retardation (GR), Plant height (PH), Grain yield (GY), Spike length (SL), Number of spikelets per spike (NSS), Number of grains per spike (NGS), Grain weight per spike (GWS), and Hundred-grain weight (HGW).

**Trait A**	**Trait B**	**ρ**	**p-value**	**Significance**

Trait A	Trait B	ρ	p-value	Significance
SMD	WH	−0.494	<0.0001	***
SMD	GR	+0.501	<0.0001	***
SMD	GY	−0.512	<0.0001	***
SMD	SL	−0.400	0.0015	**
SMD	NSS	−0.529	<0.0001	***
SMD	NGS	−0.527	<0.0001	***
WH	GR	−0.819	<0.0001	***
WH	PH	+0.534	<0.0001	***
WH	GY	+0.796	<0.0001	***
GR	PH	−0.555	<0.0001	***
GR	GY	−0.862	<0.0001	***
GY	NGS	+0.510	<0.0001	***
GY	GWS	+0.473	0.0001	***
SL	NSS	+0.882	<0.0001	***
NGS	GWS	+0.817	<0.0001	***
GWS	HGW	+0.780	<0.0001	***

Table S5. Pairwise Spearman rank correlations of snow mold damage (SMD) among the four infectious backgrounds (n = 60 accession means), with 95% confidence intervals (CI) and Holm-adjusted p-values for multiple comparisons.

Background pair	Spearman rank correlations (ρ)	95% CI	p (raw)	p (Holm-adjusted)	Significance
NIB vs EIB-1	0.43	[0.197, 0.616]	0.0006	0.0037	**
NIB vs EIB-2	0.208	[-0.049, 0.438]	0.1116	0.5578	ns
NIB vs EIB-3	-0.049	[-0.3, 0.207]	0.7081	1.0	ns
EIB-1 vs EIB-2	0.171	[-0.087, 0.407]	0.1922	0.7687	ns
EIB-1 vs EIB-3	-0.019	[-0.271, 0.236]	0.8881	1.0	ns
EIB-2 vs EIB-3	-0.048	[-0.298, 0.209]	0.7172	1.0	ns

Table S6. Eigenvalues and variance explained by the eight principal components (PC) with eigenvalue ≥ 1, retained from principal component analysis of 60 winter rye (*Secale cereale*) accessions. The analysis was based on 40 standardized variables (10 traits × four infectious backgrounds).

Principal Component	Eigenvalue	Variance Explained (%)	Cumulative Variance (%)
**PC1**	13.109	32.2	32.2
**PC2**	6.514	16.0	48.2
**PC3**	3.912	9.6	57.8
**PC4**	3.571	8.8	66.6
**PC5**	2.075	5.1	71.7
**PC6**	1.455	3.6	75.3
**PC7**	1.296	3.2	78.5
**PC8**	1.087	2.7	81.2

Table S7. Complete matrix of principal component loadings (40 variables × 8 retained components) underlying the PCA biplot (Figure 3) for 60 winter rye accessions.

Variable	PC1	PC2	PC3	PC4	PC5	PC6	PC7	PC8
SMD_NIB	-0.173	-0.032	-0.158	0.01	-0.099	-0.264	-0.04	0.132
SMD_EIB-1	-0.202	0.016	-0.087	-0.077	-0.053	-0.006	0.267	0.024
SMD_EIB-2	-0.099	-0.087	0.066	0.011	-0.307	-0.146	-0.197	0.209
SMD_EIB-3	-0.037	-0.026	0.082	0.131	0.393	-0.058	-0.099	0.497
WH_NIB	0.075	-0.158	0.112	0.302	0.056	0.13	0.376	-0.033
WH_EIB-1	0.178	-0.124	-0.023	0.142	0.135	-0.064	-0.132	0.189
WH_EIB-2	0.204	0.001	-0.173	-0.043	0.197	-0.139	0.029	-0.061
WH_EIB-3	0.179	-0.188	-0.075	-0.058	0.119	-0.232	-0.057	-0.16
GR_NIB	-0.088	0.119	0.011	-0.388	-0.134	-0.081	-0.251	-0.007
GR_EIB-1	-0.209	0.132	0.071	-0.139	-0.092	0.09	0.112	-0.081
GR_EIB-2	-0.23	-0.071	0.083	0.085	-0.113	-0.069	0.033	0.147
GR_EIB-3	-0.145	0.194	0.139	0.162	0.026	0.226	-0.063	0.264
PH_NIB	0.087	-0.283	-0.138	0.076	-0.092	0.295	0.055	0.114
PH_EIB-1	0.127	-0.256	-0.105	0.001	-0.136	0.205	-0.197	0.154
PH_EIB-2	0.149	-0.193	-0.185	-0.15	-0.119	0.3	-0.073	0.101
PH_EIB-3	0.111	-0.216	-0.208	-0.145	-0.233	0.03	-0.082	0.108
GY_NIB	0.099	-0.005	-0.069	0.417	0.167	-0.068	0.208	-0.013
GY_EIB-1	0.238	0.021	-0.121	0.103	0.081	-0.095	-0.029	0.147
GY_EIB-2	0.227	0.012	-0.185	-0.044	0.063	0.008	-0.035	-0.107
GY_EIB-3	0.17	-0.181	-0.107	-0.023	-0.086	-0.196	0.21	-0.299
SL_NIB	0.144	0.12	0.254	-0.207	-0.1	0.119	0.186	0.113

SL_EIB-1	0.189	-0.081	0.275	0.028	-0.125	0.06	-0.072	0.052
SL_EIB-2	0.193	0.005	0.239	-0.112	0.161	0.156	-0.15	0.045
SL_EIB-3	0.194	0.009	0.086	-0.217	-0.056	0.082	0.337	0.192
NSS_NIB	0.153	0.17	0.26	-0.094	-0.089	0.017	0.208	0.088
NSS_EIB-1	0.17	-0.082	0.261	0.04	-0.171	-0.131	-0.049	-0.155
NSS_EIB-2	0.193	0.075	0.223	-0.099	0.196	0.044	-0.154	-0.063
NSS_EIB-3	0.208	-0.017	0.091	-0.147	-0.091	0.035	0.291	0.152
NGS_NIB	0.099	0.261	0.071	0.103	-0.18	-0.293	0.077	-0.014
NGS_EIB-1	0.168	-0.026	0.181	0.218	-0.216	-0.172	-0.203	0.024
NGS_EIB-2	0.158	0.213	0.089	-0.015	0.128	-0.005	-0.21	-0.083
NGS_EIB-3	0.205	0.1	-0.143	-0.094	-0.077	-0.171	0.102	0.216
GWS_NIB	0.048	0.319	-0.11	0.112	-0.147	-0.144	0.057	0.118
GWS_EIB-1	0.152	0.104	0.027	0.312	-0.282	0.041	-0.132	-0.023
GWS_EIB-2	0.133	0.267	-0.051	-0.006	0.135	0.193	-0.141	-0.167
GWS_EIB-3	0.201	0.146	-0.202	-0.089	-0.069	-0.127	0.05	0.172
HGW_NIB	-0.018	0.254	-0.27	0.084	-0.094	0.11	0.044	0.208
HGW_EIB-1	0.072	0.139	-0.048	0.28	-0.326	0.272	-0.089	-0.185
HGW_EIB-2	0.022	0.241	-0.232	0.015	0.054	0.303	0.036	-0.153
HGW_EIB-3	0.136	0.21	-0.231	-0.067	0.036	-0.025	-0.075	0.073

Table S8. List of the 60 winter rye (*Secale cereale*) accessions evaluated in this study, including accession code, accession name, seed source, country of origin, and accession type (OP – open-pollinated cultivar; Hybrid – F1 hybrid cultivar; Landrace – farmer-selected population). VIR – N.I. Vavilov All-Russian Institute of Plant Genetic Resources. KSC – Kazan Scientific Center of the Russian Academy of Sciences.

Code	Accession name	Seed source	Country of origin	Accession type
G1	Nomare	VIR	Germany	OP
G2	Irina	VIR	Russia	OP
G3	Sibirskaya 87	VIR	Russia	OP
G4	VPK	VIR	Ukraine	OP
G5	Kaupo	VIR	Latvia	OP
G6	Rafle Fall	VIR	Canada	OP
G7	Pico Urugway	VIR	Uruguay	OP
G8	Jan An	VIR	China	OP
G9	Era	VIR	Russia	OP
G10	Bezenchukskaya 110	VIR	Russia	OP
G11	Vostok	VIR	Belarus	OP
G12	Lota	VIR	Belarus	OP

G13	Evrika	VIR	Russia	OP
G14	Ophelia	VIR	Belarus	OP
G15	Saratovskaya 10	VIR	Russia	OP
G16	Novosibirskaya 17	VIR	Russia	OP
G17	Grafinya	VIR	Russia	OP
G18	Kiprez	VIR	Russia	OP
G19	Flora	VIR	Russia	OP
G20	Moskovskaya 18	VIR	Russia	OP
G21	Talovskaya 45	VIR	Russia	OP
G22	Dana	VIR	Russia	OP
G23	Darvet	VIR	Russia	OP
G24	Peremena	VIR	Russia	OP
G25	Togna	VIR	Russia	OP
G26	Arga	VIR	Russia	OP
G27	Chulpan 9	VIR	Russia	OP
G28	D-3918, Domestic (Vologda)	VIR	Russia	Landrace
G29	D-8373, Domestic	VIR	Ukraine	Landrace
G30	D-8559, Domestic (Altay)	VIR	Russia	Landrace
G31	D-9034, Domestic	VIR	Belarus	Landrace
G32	D-10069, Domestic	VIR	Bulgaria	Landrace
G33	D-10392, Domestic	VIR	Serbia	Landrace
G34	D-10625, Domestic	VIR	Finland	Landrace
G35	D-10637, Domestic	VIR	Finland	Landrace
G36	D-10648, Domestic	VIR	Finland	Landrace
G37	D-11324, Domestic	VIR	Serbia	Landrace
G38	Getera 2	VIR	Russia	OP
G39	Immunnaya 6	VIR	Russia	OP
G40	Orgib	VIR	Russia	OP
G41	Xarkovskaya 55-2	VIR	Russia	OP
G42	Imerig 4	VIR	Russia	OP
G43	Sarumros 5	VIR	Russia	OP
G44	Volkhova 2	VIR	Russia	OP
G45	I-30/88	VIR	Russia	OP
G46	IL-23/94	VIR	Russia	OP
G47	I-32/92	VIR	Russia	OP
G48	Krupnozernaya 1	VIR	Russia	OP
G49	Tatarskaya 1	KSC	Russia	OP
G50	Estafeta Tatarstana	KSC	Russia	OP
G51	Radon	KSC	Russia	OP
G52	Ogonek	KSC	Russia	OP
G53	Tantana (local check)	KSC	Russia	OP
G54	Podarok	KSC	Russia	OP
G55	Zilant	KSC	Russia	OP
G56	Ritm	KSC	Russia	OP
G57	Nemchinovskaya 1	VIR	Russia	Hybrid
G58	KWS Ravo	KWS Lochow	Germany	Hybrid
G59	KWS Akbator	KWS Lochow	Germany	Hybrid
G60	KWS Vendor	KWS Lochow	Germany	Hybrid

Table S9. Long-term average winter climatic parameters at the study site (Laishev District, Republic of Tatarstan, Russia).

Parameter	Value / Range	Period / Notes
Average winter temperature	−13.6 °C to −14.8 °C	January (coldest month)
Temperature extremes	−35.0 °C to −45.3 °C	Historical absolute minimum (1979)
Annual frosty days	~160 days	Total days with subzero temperature
Stable snow cover duration	145–160 days	Late November to mid-April
Average snow cover depth	30–35 cm	Mean value across the region
Maximum snow cover depth	42–53 cm	Typically reached in March
Average ground frost depth	60–92 cm	Varies by soil moisture and snow depth
Maximum ground frost depth	100–120 cm	Observed in low-snow years
Stable 0 °C transition (autumn)	Late October – early November	Autumn-to-winter shift
Spring thaw (above 0 °C)	Mid-April	Winter-to-spring shift
Winter precipitation	225–240 mm	Cumulative for the cold-season period

Table S10. Nine-point scale (1–9) used to score snow mold damage (SMD) immediately after snowmelt and post-winter growth retardation (GR) 20-25 days later in winter rye (*Secale cereale*) based on crop damage, leaf symptoms, and tillering-shoot symptoms.

Group	Score	Crop damage (%)	Crop damage symptoms	Leaf symptoms	Symptoms of damage to tillering shoots
**R (Resistant)**	1	0–0.1	No visible damage	All leaves green	Shoots and tillering nodes healthy
	2	≤ 1	Single lesion on individual plants	Most leaves green; slight lesions on lower leaves as light brown spots	Shoots and tillering nodes healthy
	3	≤ 10	Damage affecting up to 10% of plants	Partial lesions and desiccation of lower and upper leaves; sparse mycelium	Tillering shoots green; tissue turgor unaffected
**MR (Moderately resistant)**	4	≤ 20	Individual patches of affected plants up to 20% of area	Lower and part of upper leaves affected; pink or grey mycelium present	Single shoots dead; tillering nodes intact
	5	≤ 30	Affected plants occupy about one-third of area	Leaves at all canopy levels remain green on ≥ 50% of plants; pink or grey mycelial web present; leaves and sheaths turn light to dark brown and shrivel	Lateral shoots light brown; partial loss of tissue turgor; tillering nodes intact
**MS (Moderately susceptible)**	6	≤ 60	Damage affecting up to 60% of plants	Leaves of main and lateral shoots affected on > 50% of plants; pink or grey mycelium growth; green leaves only on central shoots	Lateral tillering shoots damaged or dead; partial loss of turgor
	7	≤ 80	Partial plant mortality	Severe leaf damage and desiccation; tissues destroyed; leaves covered with pink or grey	Most shoots light brown; partial loss of turgor

				mycelium; few leaves remain green	
**S (Susceptible)**	8	≤ 90	Extensive plant death; die-off of lateral tillering shoots and part of main shoots	Severe leaf lesions; tissues destroyed; dense adhesive pink or grey mycelium	Shoots rot and turn brown; tissue turgor lost; partial root death
	9	91–100	Nearly complete plant death	All leaves affected; tissues destroyed and desiccated; almost continuous mycelial coating	Leaf sheaths, tillering nodes and roots dead

Table S11. Ideotype definition used to calculate the Multi-Trait Genotype-Ideotype Distance Index (MGIDI) for 60 winter rye (*Secale cereale*) accessions. The same optimization direction was applied to each trait under all four infectious backgrounds. The following 10 traits were assessed: Snow mold damage (SMD), Winter hardiness (WH), Growth retardation (GR), Plant height (PH), Grain yield (GY), Spike length (SL), Number of spikelets per spike (NSS), Number of grains per spike (NGS), Grain weight per spike (GWS), and Hundred-grain weight (HGW).

Trait	Ideotype Value	Rationale
SMD	Minimum (1 point)	1 = maximum resistance; 9 = complete plant death
GR	Minimum (1 point)	1 = no growth retardation; 9 = severe retardation
WH	Maximum (%)	Maximum winter survival rate
GY	Maximum (g/m²)	Highest grain yield per unit area
SL	Maximum (cm)	Longest spike
NSS	Maximum (pcs)	Most spikelets per spike
NGS	Maximum (pcs)	Most grains per spike
GWS	Maximum (g)	Highest grain weight per spike
HGW	Maximum (g)	Highest hundred-grain weight
PH	Maximum (cm)	Proxy for post-winter regrowth (positively correlated with WH, negatively with GR

Table S12. Multi-trait genotype-ideotype distance index (MGIDI) values and final ranks for all 60 winter rye accessions, corresponding to Figure 4.

Name	MGIDI	Rank
Grafinya	7.83	1
Sibirskaya 87	8.01	2
Kiprez	8.95	3
VPK	9.52	4
Chulpan 9	9.79	5
Estafeta Tatarstana	9.86	6
Flora	9.95	7

Name	MGIDI	Rank
Arga	10.02	8
Zilant	10.31	9
Tatarskaya 1	10.49	10
Tantana	10.73	11
Ritm	10.75	12
Podarok	10.85	13
Xarkovskaya 55-2	10.93	14
Getera 2	11.03	15
Radon	11.16	16
Novosibirskaya 17	11.45	17
D-8559	11.72	18
Irina	11.99	19
Bezenchukskaya 110	12.05	20
Era	12.17	21
Volkova 2	12.28	22
Immunnaya 6	12.34	23
Pico Urugway	12.35	24
Darvet	12.53	25
Ogonek	12.53	26
Moskovskaya 18	12.58	27
D-3918	13.11	28
Ophelia	13.18	29
I-30/88	13.3	30
Orgib	13.43	31
Talovskaya 45	13.57	32
Kaupo	13.6	33
Nomare	13.76	34
Rafle Fall	13.92	35
Jan An	14.19	36

Name	MGIDI	Rank
Imerig 4	14.54	37
Dana	14.55	38
Evrika	14.75	39
D-8373	14.8	40
Sarumros 5	14.88	41
Peremena	14.92	42
Saratovskaya 10	14.96	43
Vostok	15.08	44
Lota	15.24	45
Togna	15.32	46
I-32/92	15.41	47
D-9034	15.98	48
Krupnozernaya 1	16.13	49
IL-23/94	16.19	50
Nemchinovskaya 1	16.5	51
D-10648	16.82	52
D-10637	17.09	53
D-10069	17.2	54
I-11324	18.17	55
D-10392	18.7	56
D-10625	18.72	57
KWS Akbator	19.45	58
KWS Vendor	23.46	59
KWS Ravo	24.72	60

Table S14. Friedman test for differences among the four infectious backgrounds (accession means; n = 60 accessions as blocks) and pairwise Wilcoxon signed-rank comparisons with Bonferroni correction for ten traits in 60 winter rye (*Secale cereale*) accessions. W – Kendall's coefficient of concordance. NIB – natural infectious background; EIB-1, EIB-2, EIB-3 – backgrounds experimentally enriched with *Microdochium nivale*, *Typhula incarnata*, and *T. ishikariensis*, respectively. *p < 0.05, **p < 0.01, ***p < 0.001, ns – not significant.

**Trait**	**χ^2 (df**	**p**	**W**	**NIB–**	**NIB–**	**NIB–**	**EIB-**	**EIB-**	**EIB-**

	= 3)			**EIB-1**	**EIB-2**	**EIB-3**	**1–EIB-2**	**1–EIB-3**	**2–EIB-3**
Snow mold damage (SMD)	136.5	<0.001	0.76	***	***	***	***	ns	***
Winter hardiness (WH)	144.7	<0.001	0.80	***	***	***	***	***	***
Growth retardation (GR)	145.6	<0.001	0.81	***	***	***	***	**	***
Plant height (PH)	103.4	<0.001	0.57	***	ns	***	***	**	***
Grain yield (GY)	153.0	<0.001	0.85	***	***	***	ns	***	***
Spike length (SL)	34.2	<0.001	0.19	***	*	*	***	***	ns
Number of spikelets per spike (NSS)	51.5	<0.001	0.29	***	**	**	***	***	ns
Number of grains per spike (NGS)	98.2	<0.001	0.55	***	***	***	***	*	ns
Grain weight per spike (GWS)	91.4	<0.001	0.51	***	***	***	**	ns	***
Hundred-grain weight (HGW)	32.3	<0.001	0.18	***	*	***	*	ns	***
